# Experimental Approaches to Visualize Effector Protein Translocation During Host‐Pathogen Interactions

**DOI:** 10.1002/bies.202400188

**Published:** 2025-03-13

**Authors:** Verena Nadin Fritsch, Michael Hensel

**Affiliations:** ^1^ Abt. Mikrobiologie Universität Osnabrück Osnabrück Germany; ^2^ Center for Cellular Nanoananalytics (CellNanOs) Universität Osnabrück Osnabrück Germany

**Keywords:** effector, self‐labeling enzymes, T3SS, T4SS, T6SS, translocation

## Abstract

Bacterial pathogens deliver effector proteins into host cells by deploying sophisticated secretion systems. This effector translocation during host‐pathogen interactions is a prerequisite for the manipulation of host cells and organisms and is important for pathogenesis. Analyses of dynamics and kinetics of translocation, subcellular localization, and cellular targets of effector proteins lead to understanding the mode of action and function of effector proteins in host‐pathogen interplay. This review provides an overview of biochemical and genetic tools that have been developed to study protein effector translocation qualitatively or quantitatively. After introducing the challenges of analyses of effector translocation during host‐pathogen interaction, we describe various methods ranging from static visualization in fixed cells to dynamic live‐cell imaging of effector protein translocation. We show the main findings enabled by the approaches, emphasize the advantages and limitations of the methods, describe recent approaches that allow real‐time tracking of effector proteins in living cells on a single molecule level, and highlight open questions in the field to be addressed by application of new methods.

## Graphical abstract

Translocation of effector proteins is a key virulence trait of many pathogenic bacteria. Various strategies were developed to visualize the translocation of effector proteins, and this review summarizes experimental approaches for analyses of translocation towards localization, kinetics, and dynamics on single molecule level.

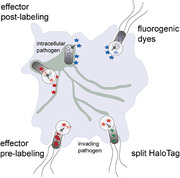

## 1 Introduction

Protein transport across cellular membranes is a fundamental property of bacteria required for motility, nutrient acquisition, quorum sensing, and so forth. As an adaptation strategy, many bacteria, including clinically relevant human pathogens, further acquired specialized translocation machineries, namely type III, type IV, and type VI secretion systems (T3SS, T4SS, T6SS)^1^ [1]. These multi‐protein complexes enable pathogenic and symbiotic bacteria to translocate proteins, referred to as effector proteins, directly into eukaryotic target cells. These effectors contribute to cell invasion, niche establishment, pathogenesis, and immune evasion required for bacterial survival and replication within hosts. T3SS, T4SS, and T6SS are essential virulence factors of various pathogens, including *Yersinia pestis, Legionella pneumophila*, and *Pseudomonas aeruginos*a [2, 3, 4]. Hence, several studies were directed to elucidate the structural and regulatory features and assembly pathways of the nanomachines (reviewed in [5, 6, 7, 8]) and to exploit them as an anti‐virulence target for therapeutic design [2, 3, 9]. Especially recent advances in (single‐particle) electron microscopy and cryo‐tomography, and 3D minimal photon flux (MINFLUX) nanoscopy have provided insights into the three‐dimensional structure of these secretion systems [10, 11]. However, the central feature of these systems, the translocation process, including the activation mechanism of translocation upon cell contact and the effector recruitment, recognition and transport through the secretion system, is still insufficiently resolved [12, 13]. Furthermore, the identification of secreted effectors, as well as their targets and functions in host cells are ongoing research areas, aggravated by experimental difficulties. These limitations include (i) the low copy number of effector proteins translocated by bacteria, (ii) the dilution of translocated effectors within the large volume of a host cell, (iii) the incompatibility of conventional fluorescent proteins, such as GFP, with the T3SS and T4SS machinery, (iv) the highly diverse biochemical nature of effectors, and (v) lack of conservation between effector of bacterial pathogens [12, 14, 15]. Consequently, sequence similarities and the relative gene localization within the genomic context can give strong indications, but the identification of (novel) effectors solely based on genomics is inconclusive [16]. Machine‐learning approaches that consider additional parameters such as codon usage, physiochemical properties and tertiary structure similarities of signal sequences facilitate more precise effector predictions [17]. Alternatively, several studies applied quantitative proteomics to identify putative effector proteins in the supernatants or associated with different host cell organelles, such as the nucleus and lipid droplets [18, 19, 20, 21, 22].

Owing to the central role of effector translocation systems for virulence, up to 10% of the coding sequence, corresponding to approximately 90 proteins, is attributed to secreted effector proteins in certain bacteria, such as *Chlamydia trachomatis* [23]. An extreme case is *L. pneumophila* with over 300 effectors that are translocated by the T4SS [24]. This complexity and high functional redundancy of effectors hampered the elucidation of their functional relationship and interaction with cellular targets in host cells. Moreover, in certain pathogens, multiple secretion systems belonging to the same class are present [5]. For instance, *Burkholderia thailandensis* encodes five T6SS, and for three of the T6SS distinct functional roles were demonstrated [25]. *Salmonella enterica* expresses two distinct T3SS, encoded on *Salmonella* pathogenicity island 1 (SPI1) and 2 (SPI2). Although SPI1‐T3SS is required for the invasion of non‐phagocytic cells, SPI2‐T3SS allows the formation of a replication‐permissive niche within host cells. Despite this functional segregation and differential expression patterns, including the reciprocal inhibition of gene expression, some effector proteins were demonstrated to be substrates for both secretion systems [26], although the molecular mechanisms are currently unknown.

Elucidating the complex interplay between effectors and host proteins should lead to a greater understanding of the overall strategies that bacteria employ to ensure survival and replication. Importantly, even effectors with very high sequence identity (>90% identity) show divergent localization patterns and functions, which in case of VFX10045 and Lpp3070 of *L. pneumophila* was attributed to two amino acid substitutions [27]. Such observations emphasize the need for detailed studies of effectors that may appear redundant due to the high level of conservation. As a direct result of the plethora of biochemical activities, including orthogonal post‐translational modifications of host proteins, effectors can interfere with various signaling cascades, implicated in a variety of biological processes, including cytoskeleton and endosomal remodeling and immune responses [28, 29]. Hereby, it appears that several effectors act in concert or antagonize each other to trigger specific host responses in a timely defined fashion [12]. Recent studies further indicate that effector proteins act dependent on the host cell context and their microenvironments, build complex functional subnetworks depending on post‐translocation modifications and cell type, and that these interactions result in distinct host responses [30, 31, 32, 33]. These findings highlight the importance of further research on effectors to understand host‐pathogen interactions and for therapeutical intervention. Hence, in recent years substantial efforts were made to study substrate identity, delivery, and hierarchy, as well as the subcellular localization, translocation dynamics, and biological functions and interaction partners throughout the entire infection process.

In this review, we summarize the available methods to study effector translocation and their contribution to our current knowledge. We will start with a short overview of simple methods that allow the detection of T3SS and T4SS effector accumulation in infected host cells at distinct time points, followed by techniques with single‐cell resolution used for the localization of effector proteins. In the last part, we will elaborate on newer methods that can be used to analyze effector protein dynamics in more detail. Hereby, we feature the recent methodological advances to study effector translocation, including spatial‐temporal characteristics in real‐time in living cells, enabled by recent improvements in microscopy. In particular, the application and potential of self‐labeling enzyme (SLE) tags will be discussed in detail.

### 1.1 The Toolbox to Study Bacterial Effector Protein Translocation Into Eukaryotic Host Cells

**FIGURE 1:**
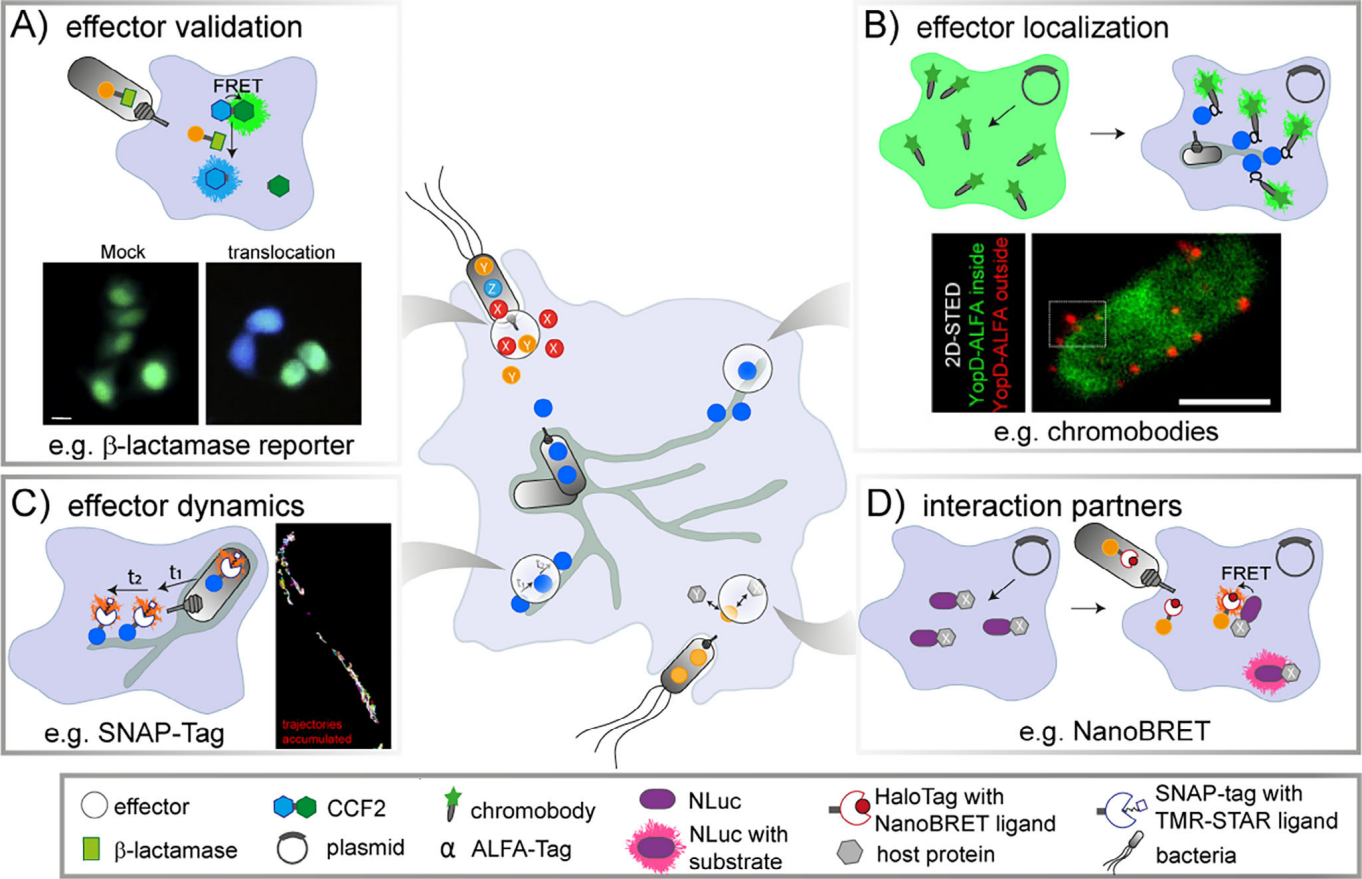
Approaches used to study different aspects of effector translocation. (A) The effector fusion with a β‐lactamase reporter is a common technique to demonstrate the translocation of a putative effector protein. Enzymatic ligand cleavage results in FRET uncoupling and light detection at 447 nm. The micrograph was adapted from Jiang et al. [234] and shows the translocation of PldA by *P. aeruginosa* into HeLa cells using substrate CCF2‐AM. Scale bar, 10 µm. (B and C) Epitope tags and self‐labeling enzyme (SLE) tags are used to analyze effector dynamics, including time‐resolved localization and translocation kinetics. Binding of plasmid‐encoded chromobodies or membrane‐permeable dyes allows effector detection at the subcellular or single‐molecule level, respectively. Single‐molecule tracking of SifA‐HaloTag fusions after TMR labeling reveals the molecular trajectories recorded within 200 consecutive frames (frame rate: 32 frames per s) as reported by Göser et al. [70]. The 2D‐STED image from Rudolph et al. [68] visualizes by differential labeling of *Y. enterocolitica* invading HeLa cells with fluorescently tagged nanobodies the cytoplasmic (green) and secreted, translocon‐associated (red) pool of YopD‐ALFA. Scale bar, 1 µm. (D) Combination of HaloTag and NLuc approaches. The enzymatic activity of NLuc generates the donor signal for the HaloTag ligand and allows co‐localization studies to identify potential interaction partners of effector proteins. Reproduced, with permission, from [234] (A), [68] (B), and [70] (C).

The analysis of effector protein translocation has benefited mainly from the advances in fluorescence microscopy, especially super‐resolution techniques for imaging beyond the diffraction limit of light [4, 34]. In fact, most of the methods currently in use, which are summarized in Table 1, require the detection of fluorescent signals. Each of these methods allows the analyses of specific aspects of the injection process, such as the identity of translocated effector proteins, their spatial distribution in bacteria and host, interaction partners, and their translocation dynamics (Figure 1). Therefore, we hope that this updated review will serve, together with the work of others [16, 35–37], as a selection guide to identify the appropriate approach depending on the research question, and to encourage further method development.

**TABLE 1:** Overview of detection methods for the visualization and analysis of effector proteins.

Method	Size	Detection method	Application	Secretion system	References
*Reporter‐based assays*					
CyaA	126 kDa	competitive immunoassay (ELISA); Western Blot analysis	Verify effector translocation	T4SS (*C. burnetii*)	Larson et al. [216]
				SPI2‐T3SS (*S*. Typhimurium); T3SS2 *(V. parahaemolyticus*) T3SS (*E. piscicida; R. solanacearum*)	Bullones‐Bolanos et al. [217]; Hu et al. [218] Liao et al. [219]; Qiu et al. [220]
			Regulation of effector translocation	T4SS (*L. pneumophila*)	Kim et al. [221]
				T3SS (*Y. enterocolitica; E. amylovora*) T3SS1 (*V. parahaemolyticus)*	Letzelter et al. [222]; Castiblanco et al. [223] Lian et al. [224]
ELK‐Tag	35 AA	Immunodetection by phosphor‐specific and ‐unspecific AB	Regulation of effector translocation	T3SS (*Y. pestis*)	Day et al. [225]
GSK‐Tag	3 kDa	Immunodetection/immunolabeling by phospho‐specific and ‐unspecific AB	Verify effector translocation	T3SS (*C. trachomatis; C. pneumoniae*)	Steiert et al. [226]; Yanatori et al. [227]
			Proof of concept	T3SS, T4SS	Garcia et al. [228]
			Effector localization	T3SS (*C. trachomatis*)	Bauler and Hackstadt 2014 [229]
CRAfT assay	38 kDa	Depending on the reporter gene, e.g., detection of GFP expression by fluorescence imaging	Verify effector translocation	T4SS (*E. coli*)	Al Mamun et al. [230, 231]
				SPI1‐T3SS (*S*. Typhimurium)	Briones et al. [232]
				T6SS (*P. aeruginosa)*	Wu et al. [233]
β‐lactamase	29 kDa	Fluorescence signal	Verify effector translocation	T6SS (*P. aeruginosa*)	Jiang et al. [234]
				T4SS (*L. pneumophila*)	Monteiro et al. [27]
				T3SS (*Y. pestis*)	Schesser Bartra et al. [40]
			Regulation of effector translocation	SPI1‐ and SPI2‐T3SS (*S*. Pullorum, *S*. Typhi) T3SS (*Y. pseudotuberculosis*)	Hamblin et al. [56]; Yin et al. [235] Gurung et al. [236]
			Translocation dynamics	SPI1‐T3SS (*S*. Typhimurium) T3SS (*E. coli*) T3SS (*Y. enterocolitica*)	Mills et al. [48, 49] Milne‐Davies et al. [42]
				T4SS (*L. pneumophila*)	Pillon et al. [108]
			Identify cell‐type specific translocation differences	T4SS (*H. pylori*) T3SS	Behrens et al. [51]; Nguyen et al. [52] Bohn et al. [50]
			Identification of secretion inhibitors	T3SS (*E. coli*)	Mühlen et al. [45]
				T4SS (*L. pneumophila*)	Cheng et al. [2]
*Epitope tags*					
FLAG	1 kDa	Immunodetection with anti‐FLAG antibody	Effector translocation	T3SS (*E. piscicida*)	Zhou et al. [60]
			Effector localization	SPI1‐T3SS (*S*. Typhimurium)	Brawn et al. [206]
				T4SS (*H. pylori*)	Andrzejewska et al. [237]
				T6SS (*K. pneumoniae*)	Sá‐Pessoae et al. [238]
		Immunoprecipitation	Identification of interaction partners	T3SS (*P. syringae*)	Lopez et al. [239]
M45	18 AA	Immunodetection with anti‐M45 antibody	Effector translocation/stability	SPI1‐T3SS (*S*. Typhimurium)	Schlumberger et al. [240]
			Effector localization	SPI2‐T3SS (*S*. Typhimurium)	Kuhle and Hensel 2002 [241]
Myc	1 kDa	Immunodetection with anti‐Myc antibody and Immunoprecipitation	Effector translocation and identification of interaction partner	T3SS (*E. coli*)	Campellone et al. [242]
		Immunodetection with anti‐Myc antibody	Effector translocation	T6SS	Wang et al. [243]
			Effector localization	T3SS (*B. pseudomallei*)	Broek et al. [244]
				T4SS (*B. abortus*)	Marchesini et al. [245]
HA	1.1 kDa	Immunodetection with Anti‐HA antibody	Effector localization	SPI1‐ and SPI2‐T3SS (*S*. Typhimurium)	Yuan et al. [59]; Yu et al. [72]; Brawn et al. [206]
				T4SS (*L. pneumophila*)	Monteiro et al. [27]
ALFA‐Tag	1.9 kDa	Fluorescent nanobodies		T3SS (*Y. enterocolitica*)	Rudolph et al. [68]
*Fluorescent proteins*					
Split‐GFP	16 AA	Fluorescence detection	Effector localization dynamics	T4SS (*A. tumefaciens)*	Li and Pan 2017 [75]
				T3SS (*P. syringae*) SPI2‐T3SS (*S*. Typhimurium) T3SS (*C. trachomatis*)	Park et al. [74]; Henry et al. [79] McQuate et al. [81] Wang et al. [80]
Chaperone‐GFP fusion	>40 kDa	Fluorescence detection	Effector dynamics	SPI1‐T3SS (*S*. Typhimurium)	Schlumberger et al. [86]
SunTag	19 AA	Fluorescence detection of eGFP‐tagged anti‐SunTag AB	Effector localization	T3SS (*S. flexneri*)	Liu et al. [89]
				T4SS (*L. pneumophila*)	Lehman et al. [90]
101B coiled‐coil peptide tag	<5 kDa	Fluorescence detection of GFP‐tagged coiled‐coil peptide 101A	Effector dynamics	NA	Gidden et al. [91]
SpyTag	13 AA	Fluorescence detection of GFP‐tagged SpyoIPD	Effector dynamics	NA	Hinrichsen et al. [246]
*Assays based on bioluminescence*					
NLuc	19 kDa (NanoLuc) 1.3 (HiBit)	Bioluminescence detection	Effector dynamics	T4SS (*H. pylori*)	Lettl et al. [88]
				SPI1‐T3SS (*S*. Typhimurium)	Westerhausen et al. [87]
			Effector translocation	T3SS (*P. syringae*)	Miao et al. [94]
				T3SS and T4SS	Fromm et al. [96]
Self‐labeling (enzyme) tags					
PYP	14 kDa	Fluorescence of fluorogenic probes		NA	Reja et al. [149]
Tetracysteine motif tag	<4.8 kDa	Fluorescence detection after FlAsH/ReAsH labeling	Effector localization	T3SS (*S. flexneri*)	Jaumouille et al. [247]
			Effector dynamics	T3SS (*S. flexneri*) SPI1‐T3SS (*S*. Typhimurium)	Enninga et al. [102] Van Engelenburg and Palmer 2008 [69]
			Effector translocation	T3SS (*B. pseudomallei*) T3SS (*P. aeruginosa*)	Treerat et al. [104] Horsman et al. [105]
				T4SS (*A. tumefaciens*)	Yaakov et al. [110]
phiLOV	13 kDa	Fluorescence detection	Effector localization	SPI1‐ and SPI2‐T3SS (*S*. Typhimurium) T3SS (*E. coli, S. flexneri*)	McIntosh et al. [130]; Patel et al. [131] Gawthorne et al. [128]
				T4SS (*A. tumefaciens)*	Roushan et al. [129]
FAST	14 kDa	Reversible binding		T3SS (*S. flexneri*)	Peron‐Cane et al. [125]
HaloTag, Clip‐Tag, SNAP‐Tag	20 ‐33 kDa	Fluorescence detection after labeling	Effector localization and dynamics	SPI1‐ and SPI2‐T3SS (*S*. Typhimurium)	Yuan et al. [59]; Göser et al. [178]; Göser et al. [70]; Franzkoch et al. [182]
TMP‐tag3	18 kDa	Fluorescence detection after labeling	Effector localization	NA	Mo et al. [191]
CRABPII	15.6 kDa	Fluorescence detection after labeling	Effector localization and dynamics	NA	Yapici et al. [248]
*Click‐chemistry*					
Genetic code expansion	NA	Fluorescence detection after labeling	Effector localization	SPI2‐T3SS (*S*. Typhimurium)	Singh et al. [63]; Singh and Kenney 2023 [195]
		Mass spectrometry after pulse‐chase labeling	Effector translocation dynamics	T3SS (*Y. enterocolitica*)	Mahdavi et al. [200]
		Mass spectrometry after photo‐crosslinking	Identification of interaction partner	SPI2‐T3SS (*S*. Typhimurium)	Li et al. [201]

### 1.2 Effector Validation by In Vivo Translocation Assays Using Enzymatic Tags

For the verification of effector translocation, different enzyme‐dependent translational reporter tag fusions are established. Some of them, such as the adenylate cyclase (CyaA) reporter gene assay or the ELK‐ and GSK‐tag rely on host proteins for second messenger production or post‐translational modifications, as indicators of effector injection (s. glossary). In contrast, expression‐based reporter systems, such as the Cre‐recombinase‐based CRAfT assay [38, 39], and the β‐lactamase assay led to the generation of a fluorescent signal, which can be easily assessed over a long time period in non‐destructive microscopy setups.

### 1.3 Translocation Studies by β‐Lactamase‐Mediated FRET Uncoupling

The antibiotic resistance factor β‐lactamase, a 32 kDa enzyme, is one of the most used reporter systems for validation of effector translocation and enabled the identification of novel effectors, secretion inhibitors, as well as regulatory mechanisms [2, 27, 36, 40–46]. In particular, β‐lactamase substrate coumarin cephalosporin fluorescein (CCF2), consisting of two fluorophores that are linked by a β‐lactam ring, has advanced studies of T3SS, T4SS, and T6SS in vivo [39, 47]. After translocation of effector‐β‐lactamase fusion proteins, hydrolysis of CCF2 or related CCF4 results in FRET uncoupling, leading to an emission shift from 520 to 447 nm which can also be detected at single cell level by microscopy or flow cytometry [48, 49] (Figure 1). This enabled the analyses of cell‐type specific translocation differences mediated by certain adhesion and host factors in cell culture and murine infection models [36, 44, 50]. For example, translocation of T4SS effector CagA was shown to be modulated by the interaction of *H. pylori* adhesin HopQ and the host cell adhesion molecule CEACAM in polymorphonuclear neutrophils, but not in bone marrow‐derived macrophages or dendritic cells [51, 52]. Importantly, β‐lactamase‐effector fusions proved useful to monitor effector translocation not only from invading bacteria but also at later steps of the infection, and by obligate intracellular bacteria, such as *C. trachomatis* [53, 54, 55]. This revealed that in contrast to the exclusive SPI2‐T3SS‐dependent translocation of SifA in *S*. Typhimurium, SifA in *S*. Typhi is translocated by SPI1‐T3SS at 8 h p.i., and by both systems at 16 h p.i. [56]. Albeit several studies used the β‐lactamase as a reporter to assess translocation efficiencies and hierarchies by time‐lapse experiments [48, 49], the detected intracellular signal intensities can differ between fusion partners due to effects on β‐lactamase half‐live, enzyme kinetics, and localization [54]. This led to alternative methods for assessing translocation dynamics more accurately and in combination with the effector localization.

### 1.4 Effector Localization Using Nanobodies

Understanding of molecular and cellular function of effector proteins during infection demands knowledge of levels and kinetics of translocation, and tempo‐spatial information on subcellular localization of effector and co‐localization with potential interaction partners [37]. Although immunolabeling represents an important technique for localization of effector proteins in the cellular environment [27, 57–60], immunostaining protocols requiring fixation provide only snapshots of complex dynamic processes. Moreover, they can introduce artifacts, including morphological changes and protein re‐localization [61, 62, 63, 64]. Therefore, epitope tags were established, such as the Pep‐Tag, the 1.4 kDa BC2/SPOT‐tag, or the α‐helical ALFA‐tag of 13 aa, which are recognized by cell‐permeable nanobodies or genetically encoded so‐called intrabodies fused to fluorescent proteins (chromobodies) [65, 66] (Figure 1). Conditionally stable chromobodies, which are degraded by the proteasome unless stabilized by target binding, were developed to increase the signal‐to‐noise ratio [67]. Recently, ALFA‐tag‐binding nanobodies (NbALFA) were used to monitor translocon formation and turnover in living host cells infected with *Yersinia enterocolitica* [68]. Within 20 min, most of the ALFA‐tagged translocon protein YopD was incorporated into the translocon and exposed to extracellular nanobodies, as revealed by the amount and intensity of fluorescence signals. The total lifespan of the YopD‐containing translocon was determined as approximately 27 min, and further studies are required to correlate the lifetime of translocons with the kinetics of effector translocation, for example as previously reported for *Salmonella* [69]. Since ALFA‐tag enabled tempo‐spatial resolution of translocon formation [68], simultaneous tracking of translocated effector proteins would provide new insights into the spatiotemporal dynamics of T3SS activity and dynamics of effector translocation.

### 1.5 Analysis of Effector Protein Dynamics Using Fluorescent Proteins

Due to the complexity and heterogeneity of bacterial infections and the essential role of effector proteins in these processes, analyzing effector dynamics on a single cell level in vivo is of interest to understanding host‐cell manipulation. Especially post‐translational modifications of effector proteins were demonstrated to influence substantially the localization, diffusion dynamics, and hence function of some effector proteins during infection [70, 71].

Fluorescent proteins such as GFP have become an indispensable research tool for studying protein localization, dynamics, and interaction partners [72]. However, while full‐length GFP is incompatible with effector translocation by T3SS and T4SS, bimolecular fluorescence complementation (BiFC) approaches enabled spatiotemporal analyses of effector delivery [73, 74]. For a split‐GFP approach, candidate effectors were fused to β‐strand 11 of GFP, a 16 aa peptide (1.8 kDa). After translocation, binding to GFP1‐10 (24 kDa) expressed by host cells led to reconstitution of GFP_comp_ and formation of the fluorophore. However, the long maturation time caused a significant delay of several hours between effector translocation within host cells [73, 75]. The use of alternative split fluorescence protein reporters with faster maturation rates and lower background fluorescence is likely to reduce such delays and enable the analyses of less abundant effector proteins without overexpression required previously [76, 77]. However, the BiFC approach is less suitable for analyzing early translocation events but rather used to study effector dynamics within different host cells at spatiotemporal resolution [75, 78–81]. More recently, split‐GFP was used to verify the efficient translocation and subsequent localization of AvrPto in the plasma membrane upon infection with *Agrobacterium tumefaciens* heterologous expressing the T3SS to increase the transformation efficiency of different plant species by T3SS effector‐dependent interference with the plant's immune system [82]. The application of different split fluorescent proteins in multiplexing experiments, such as sfCherry1‐10 and sfCherry 11 combined with split‐GFP [83, 84], might also allow the analysis of multiple effectors simultaneously.

An alternative approach to study effector translocation kinetics deployed the specific affinity of a dedicated chaperone for T3SS effector proteins. Here, GFP fusions were expressed by eukaryotic host cells [9, 85, 86]. Upon effector injection, the cognate chaperone was recruited, leading to the formation of GFP foci. Time‐lapse fluorescence microscopy was combined with quantitative analysis of total SipA levels in bacteria by Western Blots and depletion assays. The work revealed that SipA translocation starts 10–90 s after contact of *Salmonella* to host cells, and requires 100–600 s for complete translocation of the bacterial pool of SipA with approximately 6 ± 3 × 10^3^ SipA molecules per expressing bacterium. Interestingly, while the NanoLuc approach (see below) also demonstrated a fast onset of SipA translocation, SipA translocation was detected over 2 h [87]. Similarly, the T4SS effector protein CagA showed a plateau phase after 60 to 80 min instead of a rapid effector depletion after the onset of effector translocation [88]. As these data result from experimental observations in different host‐pathogen‐systems using distinct analysis tools, general conclusions about the kinetics of effector proteins translocation are not yet possible. Work towards such direct comparative analyses and general conclusions on effector translocation would require the application of the same, optimized, analyses tools to various host‐pathogen‐systems. However, this is currently elusive and future work would benefit from consensus on tools for effector translocation analyses.

Although certain chaperones are functionally interchangeable between different species and the combination of different chaperones in multiplexing experiments might help to elucidate the role of multi‐cargo chaperones in effector translocation hierarchy, for several effectors, the respective chaperones are unknown or may not exist. Also, the promiscuity of several chaperones, comprising up to ten interaction partners [85], complicates the analysis and interpretation of protein dynamics of a specific effector under native conditions. In these cases, the SunTag based on the recruitment of an in the host expressed eGFP‐tagged anti‐SunTag antibody to the SunTag‐effector fusion [89, 90], could be used. The super‐resolution approach LIVE‐PAINT could represent another alternative [91]. In this approach, the fluorescence protein is fused to coiled‐coil peptide 101A instead of the chaperone, while the effector protein is fused to the 101B coiled‐coil peptide tag consisting of five residues. The peptide‐peptide interaction, which shows a K_D_ of 200 nM, then leads to the re‐localization of the fluorescence protein and enables the analysis of the post‐translational localization of the tagged protein.

### 1.6 Luminescence‐Based Real‐Time Measurements Using the BiFC of NanoLuc

In addition to fluorescence‐based assays, the development of NanoLuc (19 kDa) expands the repertoire to bioluminescence as a detection method of effector translocation. Remarkably, while most reporter fusions reduce the translocation efficiency of the effector protein at least slightly, NanoLuc was reported to contain an intrinsic T4SS translocation signal in the N‐terminus [92]. However, based on the high signal‐to‐noise ratio and sensitivity, most recent effector translocation studies used the split‐NanoLuc system [87, 88, 93, 94]. The self‐assembling split‐NanoLuc system consists of two fragments, a larger 18 kDa fragment containing the β‐strand 1–9 (LgBiT), and the smaller β‐strand 10, Nluc10. Depending on the affinity for LgBiT, two Nluc10 variants, SmBiT (K_D_ of 190 µM) and HiBiT (K_D_ of 0.7 nM) can be distinguished [95]. When the small and large fragments assemble into an active enzyme, cleavage of substrates such as furimazine produces strong luminescence signals, which are directly proportional to the amount of reconstituted NanoLuc [87].

Using this approach, it was recently demonstrated that YopJ homologs YopP and AvrA of *Y. enterocolitica* and *S*. Typhimurium, respectively, are translocated by their cognate T3SS, but also promiscuously by the T4SS of *Bartonella henselae*. This was explained by the highly conserved VirB/VirD4‐dependent translocation signals at N‐ and C‐termini of effector proteins [96]. In contrast, ByeA, a YopJ family effector lacking the N‐terminal T3SS‐specific translocation signal, was translocated strictly T4SS‐dependent, suggesting a secondary acquisition that led to the evolutionary change in secretion system specificity.

Recently, the distinct conformation of PopD in functional translocons, whereby the Nluc10‐tagged N‐terminus is exposed to the host cytoplasm expressing Nluc1‐9, has been exploited as a marker for the functional state of the translocon [97]. The half‐maximal signal was observed within 15 min of *P. aeruginosa* infection, and the signal began to saturate after 45 min, indicating complete translocon assembly. These results suggest that the NanoLuc system provides a powerful resource to study the temporal relationship between functional translocon assembly and effector injection. Moreover, bioluminescent imaging was recently applied for analyses of subcellular localization of mammalian protein [98] and could pave the way to monitor effector localization upon translocation in living cells. Yet, the limitations of bioluminescent imaging are the sensitivity and spatial resolution that can be obtained by currently available microscopy systems.

NLuc has also been applied to analyze protein interactions. The proximity‐dependent functional complementation or NanoBRET is based on bioluminescence resonance energy transfer between the NLuc‐protein fusion and an acceptor fluorescence protein or HaloTag‐protein fusion (Figure 1) and offers potential for the study of effector‐host protein interactions [99]. Improvement of NLuc reporters is ongoing. Recently, the exchange of NLuc codon 162 by alanine was demonstrated to increase, at the expense of signal intensity, the half‐life of bioluminescence activity [100], important for long‐term monitoring of effector proteins.

### 1.7 Effector Translocation and Localization Analyses Using Self‐Labeling Tags

Studies relying on bimolecular complementation or protein recruitment are hampered by the time requirement and the necessity for transgenic host cells expressing the complementary part in the cellular compartments, where the effector protein is present and active [74]. Hence, several genetically encoded probes have been developed for direct effector labeling to permit real‐time monitoring of the entire translocation process and to assess intrabacterial heterogenicity with respect to total and secreted effector levels and the effector kinetics.

One of the first studies using self‐labeling tags for effector studies applied the fluorescein‐based biarsenical dye (FlAsH) for the pre‐ and post‐translocation labeling of effector proteins [101, 102]. In this approach, the effectors are fused to small peptide tags of 12–18 amino acids with a tetracysteine motif (4 Cys), and labeled with the 700 Da biarsenic dye FlAsH. Although the fluorescence intensity increased approximately 10^4^‐fold upon binding [103], the low signal intensity prevented live‐cell imaging of translocated effector proteins unless the signal was amplified by using triple 4Cys tag [69, 102]. Albeit an overall low signal‐to‐noise ratio, FlAsH was successfully used to identify novel T3SS effector proteins in *Burkholderia pseudomallei*, to analyze T3SS effector secretion in *P. aeruginosa* biofilms, to determine the translocation kinetics of IpaB and IpaC in *Shigella flexneri*, and the coordination of the antagonistic acting effectors SopE2 and SptP in *Salmonella* [69, 102, 104, 105]. Quantification of FlAsH fluorescence signals revealed that GEF‐mimicking and invasion‐inducing effector SopE2 was secreted at about two times higher rate than the GAP‐mimicking, invasion‐terminating effector SptP. Within the host cell, SopE and SptP are differentially affected by proteasomal degradation [106], resulting in 6.5‐fold faster degradation of SopE compared to SptP [69]. The combination of distinct secretion kinetics and differential degradation temporally change effector ratios and enable a fine‐tuned coordination of trigger invasion by *Salmonella* [69]. A hierarchical secretion delay of about 5 min to avoid functional interference of agonistic‐acting effector proteins during actin remodeling by *Salmonella* and *L. pneumophila* was also observed by β‐lactamase assays and immunostaining of intrabacterial effector levels at different time points after host‐cell attachment [107, 108]. Affinity differences of the different chaperone‐effector complexes to the T3SS [85], and the different binding affinities of multi‐cargo chaperones for their respective substrates could mediate this conserved hierarchical effector translocation.

Despite the availability of alternative tag variants and fluorescein derivatives, characterized by enhanced brightness and photostability [73, 109], only one study used the red‐shifted analog ReAsH for the monitoring of VirE2 in *A. tumefaciens* [110]. Site‐selective labeling of two different 4Cys motifs with FlAsH and ReAsH, respectively, can also be used to perform co‐localization and FRET studies [111]. Moreover, FlAsH labeling can be combined with super‐resolution microscopy (SRM) techniques, such as PALM [109], for single‐molecule localization to uncover novel spatio‐temporal effector regulatory mechanisms. However, the cytotoxicity of the biarsenic dye FlAsH and the antidote dithiol destaining reagents, required to reduce non‐specific binding, and the relatively long labeling times (<1 h) [102, 109], prevent extensive time‐lapse experiments and restrict kinetic analyses to pre‐labeled effector proteins expressed prior to host invasion.

### 1.8 Covalent Labeling to Assess Effector Dynamics

In contrast to self‐labeling tags, such as 4Cys, self‐labeling proteins provide a higher labeling specificity and simple washing steps are sufficient to reduce unspecific background. Moreover, the required dyes are less cytotoxic and often available with different characteristics, regarding their spectral properties, membrane permeability, and fluorogenicity, providing a high flexibility. Thus, in the early 2000s, several different self‐labeling protein tags were generated, including, the FAP‐tag, the PYP‐tag, the FAST, and the eDHFR/TMP tag and used for protein localization, tracking, and the analysis of protein–protein interactions [72, 112, 113].

The 14 kDa PYP tag binds fluorogenic substrates by transthioesterification [72]. Although initial reports documented relatively slow binding kinetics (∼2 h), accelerated labeling rates of a few minutes were achieved by the development of alternative fluorogenic probes, such as TMBDMA, or charge‐reversal mutations in the tag, creating PYP3R [114, 115]. In addition, the fluorescence decline due to nucleophilic exchange reactions by endogenous thiols was overcome by ketone‐based ligands that form stable thioether bonds with the PYP‐tag [116]. Moreover, recently, another membrane‐permeable, fluorogenic probe based on a cationic dye scaffold was reported [117]. Thus, further studies should examine the compatibility with effector secretion and translocation.

### 1.9 Effector Localization Analyses Using Noncovalent Fluorescent Labeling with Fluorogen‐Activating Protein (FAP) Tags

Non‐covalent labeling strategies have been developed to allow longer real‐time monitoring of effector dynamics by overcoming photobleaching rates by exchanging with new fluorogens, and thus allowing spectral flexibility within the same experiment [103, 118, 119].

FAP tags constrain the chromophore of their fluorogenic substances in a rigid conformation, leading to reduced excited state rotations and a strong fluorescence signal. Despite the availability of membrane‐permeable dyes, most studies focused on membrane proteins within SRM and single molecule tracking experiments [120, 121, 122]. FAPs were originally obtained from single‐chain antibodies and include flavin‐based fluorescent proteins, such as iLOV, and protein tags, such as the 14 kDa fluorescence‐activating and absorption‐shifting Tag (FAST). FAST was derived from the photoactive yellow protein (PYP) of *Halorhodospira halophila*, and various improved versions, such as iFAST, nanoFAST, and Click‐FAST, were reported [95, 123]. Importantly, the fluorogenic 4‐hydroxybenzylidene‐rhodanine (HBR) ligand derivatives have low toxicity and offer a wide variety of spectral properties [123, 124]. The potential of FAST for effector translocation studies was indicated by the observation that T3SS effector of *S. flexneri* can be pre‐labeled in bacteria prior to translocation into host cells [125], but studies further extending this feature are pending.

The small size, high pH stability, dye independence, and suitability for correlative light electron microscopy (CLEM) [126] render fluorescent proteins based on the light‐oxygen‐voltage (LOV) sensing domain as valuable alternatives to study effector proteins. Upon photoexcitation with blue or UV light, the light‐absorbing flavin mononucleotide (FMN) fluorophore, bound by the LOV domains, emits green fluorescence [127]. Protein fusions of phiLOV2.1, a derivative of the iLOV domain, demonstrated the compatibility with T3SS and T4SS effector translocation, but were restricted to high abundant proteins due to the three‐fold lower quantum yield compared to GFP [128, 129]. Interestingly, while effector injection occurred at one well‐defined focal point in *E. coli* and *S. flexneri* [128], SipA‐phiLOV in *S*. Typhimurium was shown to accumulate at the bacterial poles at multiple focal points prior to injection and caspase‐3 activation in macrophages and in an *ex vivo* intestinal model [130]. While photobleaching and phototoxicity due to near‐UV excitation might interfere with extensive time‐lapse studies [127, 129], Patel et al. were able to monitor the co‐localization of caspase‐3 and SifA over 8 h p.i. [131]. Additionally, advances in the field of optogenetics highlight the applicability of LOV domains to temporally control the activity of T3SS to allow systematic analysis of the temporal order of translocation and effector hierarchy [132, 133].

### 1.10 Effector Localization and Tracking on the Single‐Molecule Level Using Self‐Labeling (Enzyme) Tags

Although the repertoire is continuously expanded [134], the HaloTag and SNAP‐tag are currently the most popular SLE tags, and since they were also successfully applied within effector protein studies (Figure 1), we discuss SLE tags in more detail.

SNAP‐tag and CLIP‐tag are both derived from the human DNA repair protein O^6^‐alkylguanine‐DNA alkyltransferase (AGT) and possess orthogonal substrate specificity based on altered hydrogen bond formation capacities [135]. Whereas the active site cysteine of SNAP‐tag reacts irreversibly with the benzyl group of O^6^‐benzylguanine derivatives, O^2^‐benzylcytosine derivatives are recognized by CLIP‐tag [136]. Interestingly, the mutagenesis leading to SNAP‐tag not only optimized the labeling reaction to create a reliable SLE tag but also interfered with proteolytic degradation observed for wild‐type protein and early AGT tags by stabilizing the molecule [136, 137]. More recently, clickable SLE tags were developed to increase the amount of compatible labeling reagents without reducing the overall labeling rate [138, 139]. For example, in a two‐step reaction, the clickable SNAP‐tag reacts with a universal azide O^6^‐benzyl‐guanine‐derivate, which can further react with di‐benzo‐cyclo‐octyl based fluorophores by cycloaddition.

HaloTag is derived from the bacterial haloalkane dehalogenase DhaA from *Rhodococcus* sp. to provide a tag with enhanced binding kinetics and high specificity for ligands containing a haloalkance chain. HaloTag ligands (HTL) are covalently linked to functional entities such as synthetic fluorophores, for example, tetramethylrhodamine (TMR) [140, 141]. The substitution of His272 by Phe272 in HaloTag abolishes the base‐catalyzed hydrolysis of the alkyl‐enzyme intermediate that is formed upon the nucleophilic displacement reaction during substrate binding. This irreversible binding combined with an apparent second‐order rate constant of 2.7×10^6^ M^−1^ s^−1^ for HTL‐TMR ensures sufficient labeling even at low expression levels. Further mutagenesis increased the labeling kinetics to 1.9 × 10^7^ M^−1^ s^−1^. Hence, labeling reactions with HaloTag7 are several orders of magnitude faster than with SNAP‐tag (4.3 × 10^5^ M^−1^ s^−1^) or CLIP‐tag (1.9 × 10^4^ M^−1^ s^−1^) [142]. Additionally, HaloTag7 was demonstrated to be more specific and to form more photostable complexes compared to SNAP‐tag and CLIP‐tag [143]. Importantly, labeling of HaloTag7 occurs at a one‐to‐one ratio, allowing for accurate protein quantifications that can be easily assessed by in‐gel fluorescence scanning of SDS‐PAGE gels or epifluorescence microscope set‐ups at the single bacterial level [144].

Over the years, additional HaloTag variants, such as HaloTag9, and various commercially available substrates for HaloTag or SNAP‐tag have been developed, for example, for fluorescence lifetime multiplexing and improved single‐molecule localization microscopy (SMLM) in vivo [112, 142, 145–149]. Systematic studies demonstrated the requirement for careful analysis of the dye suitability prior to the experiment, as low cell permeability, non‐specific staining in mammalian cells, and rapid photobleaching appeared as major issues with several dyes [150]. Interestingly, while the SNAP‐tag showed higher substrate promiscuity and relatively stable labeling kinetics, the apparent rate constants of HaloTag are highly dye‐dependent, spanning more than six orders of magnitude and reaching rates near the diffusion limit for certain rhodamine substrates [151]. These differences in specificity could be attributed to the cavity of HaloTag which is two times smaller and more hydrophobic compared to SNAP‐tag [152]. These results showed that a careful choice of the organic dye conjugated to the SLE substrate is very important, even though organic dyes are brighter, more photostable, and available at a wider spectral range compared with fluorescence proteins, and are therefore favored in photon‐intensive imaging applications such as single‐molecule tracking (SMT) experiments [153]. Currently, the most commonly used dyes are TMR and the Janelia Fluor dyes, which are TMR derivates with *N*,*N*‐dimethylamino groups replaced by azetidine rings or deuterated pyrrolidine to increase the brightness and photostability [142, 154].

**FIGURE 2:**
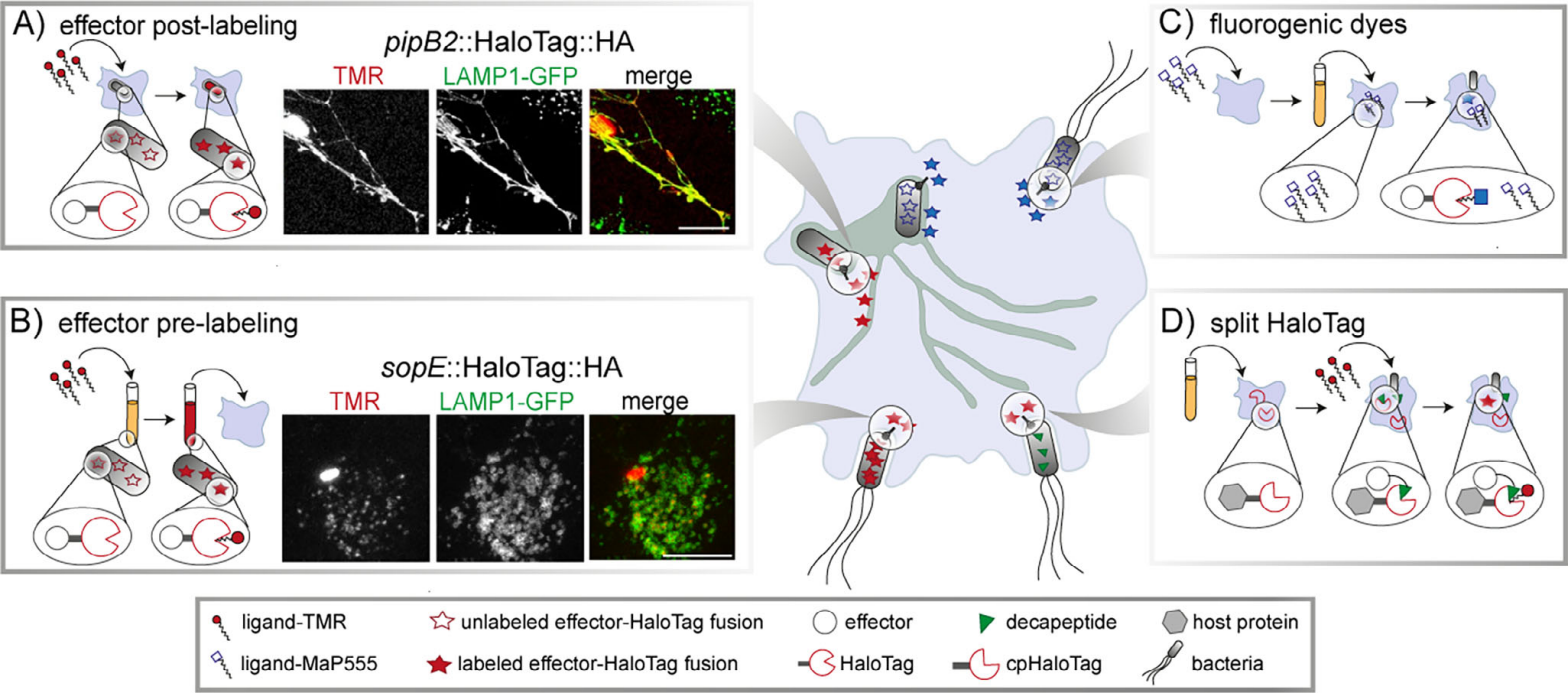
The versatility of the SLE HaloTag for visualizing effector proteins. (A, B) Cell‐permeable ligands, such as HLT‐TMR, allow labeling in living host cells or bacteria to analyze the effector translocation of intracellular (e.g., SPI2‐T3SS effectors) or invading bacteria (e.g., SPI1‐T3SS effectors). Representative post‐labeling of SPI2‐T3SS effector PipB2, adapted from Göser et al. [178], and pre‐labeling of the SPI1‐T3SS SopE are shown for *Salmonell*a invading HeLa LAMP1‐GFP cells. (C) Fluorogens show strong fluorescence only after binding to HaloTag, allowing no‐wash experiments essential to assess fast translocation dynamics. (D) The split‐HaloTag approach can be used to analyze effector‐host protein interactions. Because only a decapeptide is fused to candidate effectors rather than the entire HaloTag, there is little interference with effector translocation and function. Reproduced, with permission, from [178] (A, B).

The development of membrane‐permeable fluorogenic probes [142] allows the omission of time‐consuming washing steps to remove unbound substrates and hence background signal. This appears as a promising improvement for the assessment of fast effector translocation kinetics and molecular events directly after labeling (Figure 2). Moreover, fluorogenic probes can also help to decrease the risk of incomplete labeling due to the biosynthesis of new, unlabeled proteins after the washout of the non‐fluorogenic dye, which is particularly important when determining the relative amount of translocated effector proteins or during prolonged time‐lapse imaging where the labeled protein is diluted with each cell division [155]. Mechanistically, fluorogenic dyes are generated either by fluorophore quenching, where the quencher is part of the group removed during the labeling process or by chemical modifications, leading to an environmental sensitivity of the fluorophore or cation−π interactions between the tryptophan residue of HaloTag and the electron‐donating group of the fluorophore [156, 157]. However, the introduction of fluorescent quenchers, such as Disperse Red 1, reduced the labeling rate in several cases significantly [157]. For SNAP‐tag, quencher incorporation at the C‐8 position of the O6‐benzylguanine purine was better tolerated than at the N‐9 or N‐7 positions [158]. In contrast, environment‐sensitive dyes, which show fluorescence enhancement within the hydrophobic binding pockets of SLE tags, retained fast reaction kinetics and did not require compensatory mutations of the SLE tag for rapid labeling [159]. Upon target binding, fluorogenic rhodamine‐based fluorophores shift from a closed, non‐fluorescent but membrane‐permeable spirocyclic form to an open, fluorescent quinoid form [160]. Thereby, the fluorescence increases significantly, resulting in reduced background labeling and improved signal‐to‐noise ratios. However, compared to non‐fluorogenic dyes, environment‐sensitive fluorophores show often a lower brightness and photostability, limiting their applicability [157]. Importantly, amino acid substitutions in the vicinity of the fluorophore binding site of HaloTag not only affect reaction rates but also the optical properties of bound fluorophores, including the fluorogenicity of dimethylamino‐styrylpyridium dyes [161], indicating the potential for further improvements. Currently, Max‐Planck‐probes (MaP) represent the most fluorogenic dyes, with a reported fluorescence increase of up to 1000‐fold upon binding [162]. The conversion of a carboxyl group in rhodamine‐based dyes into an electron‐deficient amide shifts the equilibrium towards the spirolactam, resulting in a highly cell‐permeable dye. Especially for the monitoring of effectors that are expressed at later stages of infection and therefore cannot be pre‐labeled, these fluorogenic variants may allow to capture of early stages of translocation and effector dissemination. Hence, this field will benefit from continuous improvement of dyes, and development of further alternatives. For example, a fluorogenic substitute for rhodamine‐based dyes was recently created by a 5‐exo‐trig ring‐closure approach of dyes with a polymethine scaffold [163], resulting in probes pending evaluation in subsequent studies.

Several labeling probes were modified to meet the various requirements of distinct SRM techniques, such as live‐cell stimulated emission depletion (STED) microscopy and SMLM [164]. For example, recently a series of spontaneously blinking hydroxymethyl‐Si‐rhodamine (HM‐SiR) analogs based on the bright Janelia Fluor (JF) dyes with different on/off ratios to account for different labeling densities were created [165]. SRM techniques such as STED, however, benefit from non‐covalent labeling approaches for reduced photobleaching [166]. Hence, reversible variants of HaloTag were created recently by two different approaches. Although Holtmannspötter et al. [167], re‐introduced His272 for base‐catalyzed hydrolysis of the covalent intermediate and regeneration of the enzyme for subsequent labeling with an unbleached substrate, Kompa et al. [166] designed exchangeable HaloTag ligands (xHTL) by replacing the chloride with sulfonamide. Both methods led to an improvement of HaloTag technology enabling long‐term time‐lapse SRM in living cells by extended multi‐frame STED microscopy, PAINT, MINIFLUX, TALM, and SIM. Moreover, by combining two different HaloTag variants, both studies demonstrated the possibility to perform live‐cell dual‐color labeling of two different proteins. In combination with other SLE tags, such as SNAP‐tag and FAST tags [168], this would provide the opportunity to monitor the relative localization and kinetics of several effectors or effector‐host protein combinations simultaneously, providing further information on effector hierarchy and interaction partners. This multiplexing is especially facilitated by the development of SNAP‐tag mimics of fluorescent proteins, which show fast labeling rates while extending the available dye spectrum to short wavelengths, such as cyan [145]. Since only the labeling reaction of the HaloTag, and not the covalent bonds and fluorescence of the dyes are sensitive to commonly used fixatives and detergents [169], a combination with immunostaining or FAST can further increase the multiplexing possibilities.

Recently, a photo‐switchable fluorescent HTL, termed HTL–Trp–BODIPY–FF, was created for no‐wash SRM imaging in living cells [170]. The fluorogenicity of this dye is mediated by a strong fluorogenic enhancement upon binding to the hydrophobic active site of the HaloTag and subsequent steric suppression of the rotation between the phenyl group and the BODIPY core. The visualization of molecular dynamics over longer periods is facilitated by the possibility of reversible fluorescence photo‐switching of the dye by UV light irradiation. The cyclo‐reversion reaction is induced by light excitation in the visible region.

Although the application of SLE tags in procaryotes and cell cultures is well‐documented [171, 172], the continuous improvement of dyes, especially with respect to their cell permeability and fluorogenicity, also enabled the usage of this chemical labeling technology in living organisms, such as zebrafish and gastrula‐stage *Xenopus laevis* embryos and mice [173, 174, 175, 176]. Importantly, in addition to *ex vivo* labeling, different live labeling techniques were successfully employed and enabled confocal imaging of tagged proteins in vivo, which could also advance studies in the field of infection biology. However, until now, only a few studies employed SLE tags to analyze secretion systems and effector protein dynamics. By fusing HaloTag and SNAP‐tag to two components of a secretion system, namely the ATPase SiiF and the outer membrane secretin SiiC, the assembly and disassembly of the SPI4‐T1SS was visualized in real‐time at the single molecule level [177]. This revealed that approximately 36% of the expressed proteins form a functional system in the growth phase of maximal SPI4‐T1SS secretion. Recently, SNAP‐tag was used to visualize the localization of the SPI1‐T3SS effector SipA in relation to GFP‐labeled cofilin I and Atto655‐spiked G‐actin in vitro [59]. These analyses revealed that SipA binds to distinct, non‐overlapping regions and inhibits cofilin‐mediated filament severing that only occurred between bare and cofilin‐decorated segments in the absence of SipA. Moreover, the application of SLE tags proved to be highly suitable for studying the spatial‐temporal dynamics of T3SS effectors inside host cells in real‐time upon translocation [70, 178, 179]. Importantly, we have shown that effector labeling can be performed prior to the translocation event or after effector injection (Figure 2), allowing the observation of different phases of the host‐pathogen interaction. By applying SRM and co‐motion analyses, *S*. Typhimurium SPI2‐T3SS effectors were tracked with a spatial resolution of 20–25 nm. These analyses revealed bidirectional motility of SifA, SseF, and PipB2 along *Salmonella*‐induced filaments (SIF) with a velocity compared to the host transmembrane protein LAMP1 of 0.058‐0.11 µm^2^/s in HeLa or RAW264.7 macrophages [70, 178]. These effector motilities were further demonstrated to be modulated by post‐translational modifications as well as protein‐interaction partners that mediate the endomembrane targeting, as well as by the membrane properties. It was also shown that effector proteins such as PipB2 are inserted into endosomal vesicles without known prior modification by host proteins, and that endosomal vesicle harboring effector subsequently interact with other endosomal membrane compartments such as the *Salmonella*‐containing vacuole (SCV) and SIF.

SLE tags can also be used in CLEM [180, 181, 182], which would provide information on effector dynamics and ultrastructural details in the low nanometer scale at defined time points within an experiment. TMR conjugated to SLE ligands retain fluorescence during high‐pressure freezing/freeze substitution and photo‐oxidizes diaminobenzidine to an osmiophilic polymer, which is visible after osmium staining in transmission electron microscopy (TEM) sections. Recent work showed that various fluorescent SLE ligands, including TMR, remain fluorescent after osmium tetroxide fixation, dehydration, and conventional embedding in EPON. Importantly, this in‐resin CLEM approach allowed the detection of SPI2‐T3SS effector SseG‐HaloTag fusion at various compartments of the Golgi apparatus, confirming previous localization studies [183, 184].

Apart from the many advantages described above and the ongoing development of SLE tags, the considerable size of ≥20 kDa was demonstrated to interfere in certain cases with the effector function and translocation, despite the introduction of a L16 linker to reduce steric hindrance. For example, the applicability of the HaloTag was restricted to SPI2‐T3SS effectors, whereas the SNAP‐tag or CLIP‐tag proved useful for effectors of the SPI1‐T3SS of *S*. Typhimurium and the T3SS of *Yersinia* [178]. Since the molecular mechanisms for these specificities are currently unknown, careful examinations of potential polar effects on the functionality and translocation efficiency of each SLE tag/effector combination are required. Initial experiments further indicate that targeted mutagenesis of current SLE tags can improve effector translocation significantly (unpublished results). Another alternative that could enhance the translocation of the fusion protein is the usage of split‐variants of the SLE tags [185]. The self‐reassociation of the N‐terminal fragment and the C‐terminal fragment restores the enzymatic activity and would allow the detection of effector proteins and protein‐protein interactions, similar to those described for split‐GFP [73], but without the drawbacks of fluorescent proteins described above [186, 187]. As an alternative to direct fusion of the split protein fragment to the effector of interest, a tag assisted split enzyme complementation (TASEC) approach has been developed, which uses short peptide tags to mediate split protein complementation [188]. Hereby, the HaloTag fragments are fused to two binders that interact with two orthogonal, short peptide tags. These peptide tags are fused to the protein of interest, bringing the HaloTag fragments into proximity and initiating complementation. The combination of split‐CLIP‐tag and split‐SNAP‐tag further enables the simultaneous tracking of both proteins after the transient complex formation [189], allowing in principle the analyses of post‐effector‐host protein interaction events in real‐time. Complementary to this approach, a recently developed reversible split‐HaloTag system derived from circularly permuted HaloTag, facilitates the observation of dynamic and transient protein‐protein interaction processes [190] (Figure 2). This reversible split‐HaloTag consists of a folded domain and a decapeptide and shows fast reconstitution and labeling rates comparable to the SNAP‐tag. Moreover, the alternative SLE tag TMP‐tag3 was demonstrated to be superior in tagging certain mitochondrial proteins that mislocalized after fusion with HaloTag [191]. However, the proximity‐induced addition between the 18 kDa monomeric eDHFR:L28C fused to the protein of interest and the ligand TMP‐acrylamide‐conjugates is comparably slow, requiring further optimizations.

Recently, another HaloTag variant, termed BenzoHTag, was designed [192]. Based on its specificity to fluorogenic benzothiadiazole dyes, this tag also allows orthogonal labeling in combination with HaloTag7. Importantly, the live‐cell imaging using the BenzoHTag revealed improved brightness, sensitivity, reaction rates, and reduced background fluorescence in no‐wash experiments. Whether this tag is also sufficiently translocated by secretion systems and can be applied for effector translocation studies needs to be assessed in further studies.

### 1.11 Genetic Code Expansion for Analyses of Effector Translocation

All of the above‐mentioned effector fusions lead to significant size increases, which may interfere with effector translocation, localization, post‐translational modifications, or protein interactions. Moreover, these labeling approaches introduce an offset of approximately 3–10 nm between the effector protein and the fluorescent label, limiting the localization accuracy. Genetic code expansion represents an alternative approach with minimal structural and functional perturbations compatible with fluorescence nanoscopy [193, 194]. The site‐specific, co‐translational incorporation of non‐canonical amino acids requires a subsystem, comprising the exogenous aminoacyl‐tRNA synthetase, the cognate tRNA, and the non‐canonical amino acid. Different click chemical labeling procedures, such as strain‐promoted inverse‐electron–demand Diels–Alder cycloaddition (SPIEDAC), mediate the rapid reaction of the bio‐orthogonal handle of the non‐canonical amino acid and the organic fluorophore or fluorogen [63, 195]. Alternatively, fluorescent non‐canonical amino acids can be used. In 2021, genetic code expansion was applied to study the localization of the low‐abundant proteins SsaP and SifA, which were previously shown to be affected by N‐ and C‐terminal epitope tags [63, 196]. Recently, Singh and Kenney also demonstrated the possibility of combining genetic code expansion with dSTORM for improved localization of SseJ below the diffraction limit [195].

Bio‐orthogonal non‐canonical amino acid tagging (BONCAT) combined with proteomics was recently used to identify novel effectors and effector‐host interactions in various bacterial species [197, 198]. In contrast to genetic code expansion, BONCAT only requires a mutated methionyl‐tRNA synthetase variant for incorporation of methionine surrogates, such as azidonorleucine (Anl), into newly synthesized proteins during infection [199]. Pulse‐labeling with Anl in combination with affinity enrichment was used to assess differences in effector translocation between intra‐ and extracellular bacteria, and to track the translocation order of eleven effectors during *Y. enterocolitica* infections [200]. The use of photo‐ANA instead of Anl further enabled the identification of eukaryotic interaction partners by photo‐crosslinking. Despite the confirmation of kinesins, as previously established interaction partners of PipB2, this study identified flotillin as a further PipB2 target during the late‐stage of infection, providing a mechanistic explanation for the localization of PipB2 in lipid rafts [201].

## 2 Outlook

**FIGURE 3:**
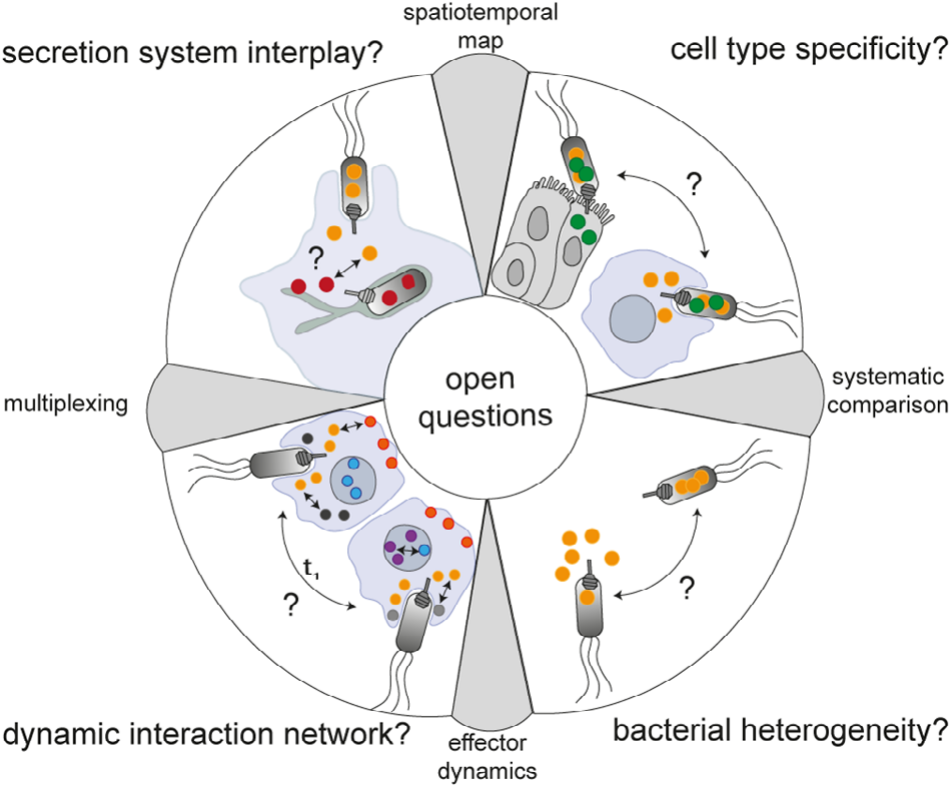
Open questions in the field that can be addressed by state‐of‐the‐art effector visualizing approaches. Systematic analyses of the effector kinetics, for example using SLE tags, combined with multiplexing approaches to identify interaction partners, are required to establish the complex and dynamic interaction networks and to assess the interbacterial variance. These networks will not only help to identify key effectors and functional redundancies but, also to clarify whether effectors of different secretion systems, such as SPI1‐ and SPI2‐T3SS, interact with each other to establish a replication niche within the host. Hereby, the consideration of different model systems is obliged to identify sub‐networks that are essential for bacterial survival, depending on the cellular context and environmental niche that the bacterium encounters. The identification of host and bacterial factors responsible for the observed heterogeneity in the effector translocation process might establish new drug targets and interference possibilities.

Despite considerable progress, particularly in the methods available, several aspects of the host‐pathogen interplay involving effector proteins, such as the complex and dynamic interaction networks, are still incompletely understood (Figure 3). Although in several cases the role of the effector is established, the coordination of their activity remains unknown. For instance, it is currently unclear whether effector proteins, such as SopE, SopE2, or SopB, which target directly or indirectly the same Rho family GTPases [202], differ in their dynamics of translation and interaction. Combining the information obtained from the many (multiplexing) options for effector localizations outlined above, and the pulse‐chase labeling technique based on the HaloTag [203, 204], in a spatio‐temporal map will help to identify redundancies, effector lifetimes, potential effector interactions, molecular targets, and interference possibilities. Moreover, it will help to elucidate the degree of crosstalk between different secretion systems involved in the trans‐kingdom delivery of bacterial effectors. For example, based on the observed co‐regulation and similar expression kinetics, a functional interaction between the SPI1‐T3SS and SPI4‐T1SS in *S. enterica* was hypothesized but remains unresolved [5, 205]. Also, the potential interplay of SPI1‐ and SPI2‐T3SS effectors, which are injected at distinct time points during the infection process, was only partially addressed in the early 2000s by demonstrating that the SPI1‐T3SS effector protein SipA was still present 8–12 h p.i. [206, 207], mediating the stabilization of SCV‐associated F‐actin, required for the proper localization of SifA and PipB2.

Single‐cell analyses revealed the bacterial heterogeneity in effector translocation, albeit the underlying mechanisms are currently unknown. For example, it was described that only a small subpopulation of *Salmonella* synthesizes effector proteins at detectable amounts [69, 86] and that the number of secretion systems per bacterium varies considerably [208, 209, 210, 211]. Bacterial heterogeneity in the availability and activity of secretion systems among bacteria most likely explains the diverse translocation efficiencies within the population reported by Mills et al. [48], determining, in combination with the innate immune response, the subsequent bacterial cell fate [37, 212]. Although some initial studies were dedicated to assessing translocation kinetics and relative effector levels [48, 49, 69, 86], systematic analyses comprising the whole set of effector proteins present within a bacterium are currently missing. However, such studies are required to understand the differences between chaperone‐dependent and ‐independent translocation dynamics, and for the comparisons between translocation systems.

So far, most studies that analyzed effector dynamics in more detail used immortalized, undifferentiated cell lines, such as HeLa cells. However, previous studies identified several cell‐type‐specific differences in effector translocation and subsequent localization patterns, reflecting the different roles executed by effector proteins depending on the cellular context [24, 37, 76]. Systematic comparisons between different cell types as well as alternative in vitro and *ex vivo* infection models, such as organoids, scaffold‐based 3D models, and precision‐cut tissue slices, that recapitulate the cellular complexity and tissue‐specific microenvironment [213, 214], should be the next step to analyze context‐dependent timing, order, quantities, localization, and network dynamics of effector injection. Microfluidic systems that allow the investigation of long‐term or even stable host‐bacterial interactions represent a promising alternative to static culture conditions, which are often characterized by rapid bacterial overgrowth [213]. These studies could benefit from recent advances in self‐driving, multi‐scale microscopy techniques that allow the observation of living spheroids or organisms while simultaneously imaging sub‐cellular dynamics [215].

## 3 Conclusions

As outlined in the text and summarized in Table 2, each of the discussed methods, including the more recently developed state‐of‐the‐art techniques, has its inherent strengths and limitations. Especially those methods, such as SLE tags, that can capture the dynamic nature of effector proteins are a key step toward understanding the bacteria‐host interplay. However, based on the provided examples, it became clear that currently most methods are limited to abundant effectors and effectors with highly defined subcellular localizations. Hence, the main challenge will be to improve the sensitivity of the methods further for the analysis of low‐abundant and highly dispersed effector proteins. Hereby, the optimization of the translocation efficiency of effector‐fusion proteins in conjunction with the development of brighter reporters/dyes with high specificity seems to be necessary. Moreover, follow‐up studies are required to exclude downstream effects on effector stability, and protein interactions. At the same time, the rapid introduction of new methods, and technological advancements, especially in the microscopy field, need to be considered and evaluated in view of their applicability to capture the dynamic processes of effector proteins.

**TABLE 2:** Comparison of various methods for labeling effector proteins.

Detection method	Working principle	Advantages	Disadvantages
CyaA	The effector of interest is genetically fused to the calmodulin‐dependent adenylate‐cyclase domain of CyaA. Upon translocation, CyaA is activated by calmodulin and converts ATP to cAMP, which can be measured using enzyme‐linked immunosorbent assay kits	High‐throughput screens possible Use of AB enables localization studies in fixed cells Only translocated effectors are detected Low interference with effector translocation	Cell lysis required → no live‐cell imaging
ELK‐tag	The ELK‐tag is genetically fused to the effector of interest. Upon translocation, the protein fusion is targeted to the nucleus by the tag's nuclear localization sequence and subsequently phosphorylated by protein kinases. Phosphospecific antibodies can be used for detection	Distinct detection of translocated vs. intra‐bacterial effectors	Cell lysis required → no live‐cell imaging Bacterial nucleotidyl cyclases interfere with the assay Interfere with native effector localization
GSK‐tag	The peptide tag is genetically fused to the effector of interest. Upon translocation, the tag is phosphorylated by cytosolic protein kinases, which can be detected by phosphospecific antibodies	Distinct detection of translocated vs. intra‐bacterial effectors Effector localization can be detected	Cell permeabilization or cell lysis required → no live‐cell imaging
CRAfT assay	The coding sequence of the Cre gene is fused to the effector of interest. Upon translocation, Cre‐mediated recombination at the lox‐sites leads to the excision of the floxed DNA sequence located between the coding region of a reporter gene and its promoter sequence, and subsequent expression of the reporter gene	Independent of dye Only translocated effectors are detected Proven compatibility with T3SS, T4SS, and T6SS	Time delay between translocation and signal detection due to GFP expression and maturation time
β‐lactamase	Genetically encoded effector‐ β−lactamase fusions are translocated into host cells, where the β−lactamase catalyzes the hydrolysis of the substrate. Substrate hydrolysis results in the separation of the two fluorophores and thus FRET uncoupling, detectable by an emission shift	Population and single‐cell analyses possible High‐throughput screens possible Early and late effector translocation can be analyzed	Signal intensities influenced by enzyme kinetics Signal is not retained at effector site → no localization studies
Epitope tags	Epitope tags are genetically attached to either the N‐ or C‐terminus of the effector of interest. Immunostaining is performed to detect the effector in fixed cells, while Western blots can be used for (semi‐)quantitative analysis	Signal amplification allows detection of low‐abundant effectors Quantification of effector abundance by WB possible Low interference with effector translocation Multiplexing possible Live‐cell imaging if nanobodies are used	Cell fixation and permeabilization are required if AB is used→ no live‐cell imaging
Split‐GFP	GFP11 is fused to the effector of interest, while GFP1‐10 is expressed in the host cell. Upon translocation, the two fragments spontaneously reassemble to form a functional GFP	Low interference with effector translocation Real‐time kinetics after the translocation No staining required	Long maturation times (>2 h) Transgenic host cells required Low abundant proteins not detectable Limited multiplexing possibilities
Effector‐chaperone interaction	The untagged effector protein is translocated into transgenic host cells. The host cells express GFP‐tagged chaperones that are specific for the effector of interest and are recruited to the effector, resulting in formation of GFP foci	Real‐time kinetics after the translocation and chaperone recruitment No staining required Wide range of fluorescence proteins	Likely interference with effector function Transgenic host cells required Limitation to an effector subset Limited specificity depending on chaperone (multicargo)
NanoLuc (NLuc)	The effector of interest is fused to HiBit, while LgBiT is expressed in the host cell. Upon translocation, the fragments assemble into a functional NLuc luciferase that generates bioluminescence upon substrate addition	Small label size → low interference with effector translocation Real‐time kinetics Co‐localization studies possible	
LOV	The bacterial effector‐light‐oxygen‐voltage (LOV) sensing domain is genetically fused to the effector of interest. Upon translocation, FMN fluorophores from the host cell bind to the LOV domain, resulting in fluorescence emission upon excitation	SRM and CLEM possible no staining required optogenetics possible	Near‐UV excitation (photobleaching, phototoxicity) Low quantum yield → low abundant proteins not detectable
PYP‐tag	The PYP‐tag is genetically fused to the effector of interest. After translocation, labeling reactions with fluorogenic substrates involve a transthioesterification reaction	Improved variants show fast labeling kinetics	Limited range of available probes Compatibility with T3SS, T4SS and T6SS not demonstrated yet
FAP tags	FAP tags are genetically fused to the effector of interest. Upon translocation, the binding reaction of the fluorogenic substance results in an increased fluorescence signal	Low background fluorescence Super‐resolution microscopy possible Fast maturation time High brightness Low cytotoxicity Variety of spectral properties	
FlAsH	A tetracysteine motif (4Cys) is genetically fused to the effector of interest. Labeling with the biarsenic dye FlAsH, followed by destaining with antidote dithiol reagents to reduce non‐specific binding, allows visualization of the effector by various microscopy techniques	Small label size Super‐resolution microscopy possible Detection of real‐time kinetics Co‐localization studies possible	Low signal intensities → low abundant effectors cannot be visualized Photo‐bleaching requires re‐labeling in long‐term experiments Limited dyes with different spectral properties Destaining reagents required Cytotoxicity of dye and destaining reagents Relatively long labeling time (<1 h)
SLE tags (HaloTag, SNAP‐tag, CLIP‐tag)	SLE‐tags are genetically fused to the effector of interest. Labeling the effector protein before or after effector translocation allows the microscopic analysis. Depending on the labeling reagent, washing steps may be required to reduce non‐bound ligands	Covalent and reversible labeling possible SRM and CLEM possible Wide variety of dyes, including fluorogenic dyes Fast labeling kinetics Detection of real‐time kinetics Effector quantification possible Multiplexing possible Co‐localization studies possible	Large tag size Partial interference with effector translocation
Genetic code expansion	Non‐canonical amino acids containing a clickable functional group are incorporated into the effector of interest by a specific orthogonal aminoacyl‐tRNA synthetase/tRNA pair during translation. Labeling by click chemistry allows visualization of the effector protein using microscopy techniques	Minimal structural and functional disruptions Super‐resolution microscopy possible Wide range of fluorescence proteins Applicable to low abundant effectors	Unspecific labeling possible High metabolic burden

In summary, the decades of research on secretion systems and the delivery of bacterial virulence factors into host cells highlight the difficulties associated with this interesting field of study. However, we envision that the summary of the applied methods, together with the outline of their strengths and limitations, will inspire researchers to improve this toolset further. Moreover, we are optimistic that especially the latest developments to track effector molecules on the single molecule level will empower us to uncover the timing and kinetics of effector protein translocation, their subcellular localization, and interaction partners to ultimately understand their functional role in establishing different intracellular lifestyles and host responses, leading to the observed heterogeneity in the context of infections. As these techniques become commonplace, we also hope that the spectrum of bacteria and serovars currently being studied for effector translocation will be expanded to provide a comprehensive understanding of the great diversity of the effector pool used by bacteria and to recognize similarities, differences, and evolutionary principles that will help us to identify potential drug targets.

## Author Contributions

VF and MH collected data, wrote and edited the original manuscript and the revised version.

## Conflicts of Interest

The authors declare no conflicts of interest.

## Supporting information



Supporting information

## Data Availability

Data sharing is not applicable to this article as no datasets were generated or analyzed during the current study.

## Endnotes

1 Please refer to Supporting Materials for a glossary defining acronyms and key bacterial and host factors.
