# Supplementary material for: Experimental Approaches to Visualize Effector Protein Translocation During Host‐Pathogen Interactions
Source: Bioessays. 2025 Mar 13;47(4):e202400188. doi: 10.1002/bies.202400188 (PMC11931682; doi:10.1002/bies.202400188)
Supplement: Supplementary file 1 — Supporting information [file BIES-47-e202400188-s001.docx]

**Glossary**

AGT: O^6^-alkylguanine-DNA alkyltransferases (AGT) are widely distributed DNA repair proteins that remove alkyl groups located at the *O*^6^-position of guanine within an irreversible self-alkylation reaction, requiring an active site cysteine. Engineered enzyme variants have been developed by the group of Kai Johnsson for the use as protein tags, such as SNAP- and CLIP-tags, which can be specifically labeled by chemical ligands.

ALFA-tag: The ALFA-tag is a rationally designed small hydrophilic, at physiological pH uncharged epitope tag that forms a stable alpha-helix recognized by high-affinity nanobodies (NbALFA).

Anl: The methionine surrogate azidonorleucine (Anl) is an unnatural amino acid used in combination with a mutant methionyl tRNA synthetase for bio-orthogonal or fluorescent labeling of newly synthesized proteins.

Atto655: Atto dyes are a series of fluorescent dyes covering the visible and near-infrared emission wavelengths and are used for FRET applications and single-molecule detection. The oxazine dye Atto 655 is a red-absorbing fluorescent dye with a decay time of approximately 1.9 ns.

AvrA: AvrA is an effector protein of *Salmonella enterica* encoded on the *Salmonella* pathogenicity island 1 that suppresses the host inflammatory response and apoptosis.

AvrPto: The infection-promoting T3SS effector protein AvrPtO of *Pseudomonas* *syringae* suppresses pathogen-associated molecular pattern (PAMP)-triggered immunity of plants by interfering with pattern recognition receptor complexes.

BC2/SPOT-tag: The SPOT-tag was developed using a rational design approach, resulting in four out of twelve amino acid substitutions of the linear peptide epitope tag BC2, which was derived from ß-catenin, to improve the binding affinities for the labeling with bivalent nanobodies.

BenzoHTag: A yeast surface display screening assay was performed to identify a HaloTag variant with improved reaction rates with a fluorogenic benzothiadiazole dye for no-wash experiments. The resulting BenzoHTag is orthogonal to the HaloTag7-rhodamine system and is characterized by improved brightness and labeling kinetics.

BiFC: The Bimolecular fluorescence complementation (BiFC) is based on the reconstitution of a functionally active fluorescent protein, such as GFP, when the two non-fluorescent protein fragments tethered to potential protein interaction partners are brought into proximity by protein-protein interactions.

BODIPY: Difluoroborondipyrromethene, commonly known as BODIPY dye, was first synthesized in 1968 and since then BODIPY derivatives have been widely used in fluorescence imaging, mainly due to their high quantum yield and tunability of photophysical properties.

BONCAT: Bioorthogonal non-canonical amino acid tagging enables the unbiased visualization and identification of the *de novo* synthesized proteome by introducing non-canonical amino acids containing a small bioorthogonal reactive group for subsequent labeling by click chemistry during protein translation.

ByeA: The T4SS-dependent *Bartonella* YopJ-like effector A (ByeA) of *Bartonella taylorii* was shown to interfere with pro-inflammatory host responses by targeting MAP kinase signaling.

CagA: The cytotoxin-associated gene A (CagA) acts as a pro-oncogenic scaffold protein in animal cells and represents a key virulence factor of *Helicobacter pylori* that is translocated into host cells by the T4SS to interfere with host immune surveillance and pro-apoptotic signaling.

CCF2: coumarin cephalosporin fluorescein (CCF2) is a fluorescent substrate of β-lactamases and consists of a cephalosporin core attached to two fluorophores that form a FRET pair, resulting in energy transfer from the 7-hydroxycoumarin donor to the fluorescein acceptor, visible as green emission. Following β-lactamase-mediated hydrolysis of CCF2, the fluorophores are separated from each other, abolishing FRET and changing the fluorescence emission spectrum from green (520 nm) to blue (447 nm).

Cdc42:  The cell division cycle 42 (Cdc42) protein is a small GTPase of the Rho family that is involved in cell cycle progression, cell polarity, actin cytoskeleton organization, and membrane trafficking, among other functions.

CEACAM: Carcinoembryonic antigen-related cell adhesion molecules (CEACAM) belong to a group of mammalian immunoglobulin-related glycoproteins and are involved in various processes, including cell-cell recognition, cell differentiation, and T-cell proliferation. Additionally, CEACAM act as receptors for host-specific viruses and bacteria.

Chromobody: Nanobodies conjugated with fluorescent proteins or organic dyes are called chromobodies.

CRAfT assay: The Cre Reporter Assay for Translocation (CRAfT) is based on the Cre/*lox* site-specific recombination system of bacteriophage P1 to visualize the translocation of Cre-effector fusions upon Cre-mediated excision of the *lox-*flanked DNA segment that prevents expression of a reporter gene in host cells.

CLEM: Correlative light electron microscopy (CLEM) combines fluorescence light microscopy, which allows the analysis of protein dynamics and the screening for rare events, with electron microscopy for high-resolution imaging to reveal the cellular ultrastructures at the very same structure observed by light microscopy.

CLIP-tag: The CLIP-tag is a 19.4 kDa self-labeling protein that represents a modified version of the SNAP-tag and reacts covalently with benzylcytosine derivatives.

Cofilin 1: The actin-interacting protein cofilin 1 is one out of two cofilin isoforms that are essential regulators of actin dynamics and function.

CyaA: The calmodulin-dependent adenylate cyclase from Bordetella pertussis, which converts adenosine triphosphate to cyclic adenosine monophosphate (cAMP), has been used as a reporter for effector translocation since 1994.

dSTORM: Direct stochastic optical reconstruction microscopy (dSTORM) is a single-molecule localization-based super-resolution microscopy method with an optical resolution of ~20 nm. It uses photoswitchable fluorophores to separate single molecules in space and time to provide a list of single-molecule coordinates for reconstructing a final image.

eDHFR/TMP-tag: The self-labeling enzyme tag eDHFR/TMP-tag is based on the high nanomolar affinity of the inhibitor trimethoprim (TMP) for the small protein domain of the *Escherichia coli* dihydrofolate reductase (eDHFR), fused to the effector of interest. Different engineering strategies, such as (TMP)-acrylamide conjugates and the introduction of a cysteine residue into eDHFR, enabled covalent labeling.

ELK: The ELK-tag consists of 35 amino acids derived from the large tumor antigen nuclear localization sequence of the simian virus 40 and the amino acids 375 to 392 of the eukaryotic transcription factor Elk-1, resulting in nuclear localization of the ELK-tagged effector, and host kinase-dependent phosphorylation, detectable by phosphospecific Elk-1 antibodies.

Epitope tags: Epitope tags are short peptides attached to the N- or C-terminus of a protein that can be recognized by specific antibodies.

EPON: The aliphatic resin EPON is one of the most widely used embedding media for electron microscopy, providing a firm base for tissue sectioning and preserving intracellular ultrastructures.

F-actin: Globular actin monomers are an essential component of the cytoskeleton and form polarized, highly dynamic, filamentous polymers, called F-actin.

FAP-tag: Originally derived from single-chain antibodies, fluorogen-activating protein (FAP) tags are based on binding-mediated fluorescence activation of otherwise non-fluorescent small molecule dyes.

FAST: The small monomeric fluorescence-activating and absorption-shifting Tag (FAST) of 14 kDa was derived from the photoactive yellow protein (PYP). The FAP-tag FAST and its variants bind non-covalently fluorogenic hydroxybenzylidene rhodanine (HBR) analogs with different spectral properties, leading to a stabilization of their fluorescent state.

FlAsH: The membrane-permeable dye fluorescein arsenical hairpin binder (FlAsH) is a fluorescein derivative used to covalently label tetracysteine-tagged proteins with high affinity.

FRET: Fluorescence resonance energy transfer (FRET) is a distance-dependent process in which non-radiative energy transfer from an excited fluorophore donor to a ground state acceptor fluorophore occurs by dipole-dipole coupling.

FMN: Flavin mononucleotide, derived from riboflavin, is an important cofactor in many biochemical reduction-oxidation reactions due to the ability of the tricyclic isoalloxazine ring system to adopt different redox states.

G-actin: The evolutionary conserved monomeric actin (G-actin) protein has a molecular weight of 42 kDa and is in a polymerization-competent state with binding sites for head-to-tail interactions with two other actin monomers to form the actin cytoskeleton.

GAP: GTPase-activating protein

GEF: guanidine nucleotide exchange factor, activator of sm

GFP: The 27-kDa monomeric green fluorescent protein (GFP) was originally isolated from Aequorea Victoria and emits, upon excitation at 488 nm, green light with an emission maximum of 509 nm. Several variants of this barrel-shaped protein which is composed of eleven ß-sheets with the chromophore at the center of the structure, have been created to modulate fluorescence intensities and spectral properties.

GSK-tag: The glycogen synthase kinase (GSK) tag consists of 13 amino acids derived from the human GSK-3β kinase. Since the GSK-tag is phosphorylated by eukaryotic kinases, phosphor-specific GSK-3β antibodies can be used to visualize the GSK-tagged effector protein after translocation into host cells.

HaloTag: The self-labeling enzyme tag HaloTag is derived from the bacterial haloalkane dehalogenase DhaA from *Rhodococcus* sp*.* and reacts with ligands containing a haloalkane chains. Different HaloTag variants and ligands allow covalent and reversible labeling reactions to visualize the effector of interest.

HBR: Cell-permeable and fluorogenic 4-hydroxybenzylidene-rhodanines (HBR) are available with different spectral properties, but while they are weakly fluorescent in solution, they strongly increase fluorescence when immobilized and conformationally locked within the protein tag FAST.

HeLa: The epithelial-like cell line HeLa was isolated in 1951 from a human cervical carcinoma and is currently one of the most widely used immortal model cell lines.

HiBiT: HiBiT is a small, 1.3 kDa tag that spontaneously associates with its complementation partner LgBit, to form a functional NanoLuc enzyme that generates a bioluminescent signal in the presence of its substrate furimazine.

HopQ: The surface-exposed outer membrane protein HopQ of *Helicobacter pylori* binds to host cell receptors, facilitating host cell adherence and translocation of virulence factors.

HM-SiR: The spontaneously blinking fluorescent probe hydroxymethyl-Si-rhodamine (HM-SiR), which transitions between an excitable quinoid and a non-excitable spiroether isomer, is based on the far-red fluorescent silicon rhodamine scaffold and has been developed for single-molecule localization microscopy in living cells.

HTL: HaloTag ligands (HTL) consist of a chloroalkane that is fused to a functional tag, such as a rhodamine-based dye. Covalent bond formation between the HaloTag protein and the chloroalkane within an HTL occurs rapidly under physiological conditions.

Intrabody: Intrabodies are functionally active intracellular antibody fragments or nanobodies. Unlike in conventional immunolabeling methods, the intracellular expression of these intrabodies allows for real-time and live-cell imaging without the destructive and invasive measures typically required to deliver conventional antibodies into a cell.

IpaB: The 62 kDa invasion plasmid antigen B (IpaB) of *Shigella* is part of the T3SS translocon structure and functions as an effector protein involved in cell entry, phagosome escape, induction of apoptosis, and release of proinflammatory cytokines.

IpaC: Together with IpaB, the invasion plasmid antigen C (IpaC) forms the translocon of the T3SS of *Shigella flexneri* and is essential for bacterial cell entry by mediating actin polymerization.

JF: Janelia Fluor dyes. Replacement of the *N*,*N*-dimethylamino groups of TMR with azetidine rings by the Lavis laboratory at Janelia Research Campus led to the creation of Janelia Fluor (JF) dyes, characterized by improved brightness and photostability. Further modifications, such as the replacement of the hydrogen atoms on the N-alkyl groups with deuterium (JFX dyes), produced a range of compounds with different spectral properties, fluorogenicity, cell permeability, and blinking characteristics.

LAMP1: The lysosomal associated membrane protein 1 (LAMP1) mainly localizes to lysosomes and late endosomes, but also to *Salmonella*-containing compartments. This heavily glycosylated lysosomal membrane protein, consisting of a large luminal, a transmembrane, and a short C-terminal cytoplasmic domain, functions in lysosome biogenesis, lysosomal pH regulation, autophagy, and cholesterol homeostasis.

LgBiT: The larger, 18 kDa fragment of the split-NanoLuc system binds HiBiT with a dissociation constant of 0.7 nM, forming the functional protein, which, in the presence of its ligand, generates a bioluminescence signal that is proportional to the amount of reconstituted and accumulated protein.

LIVE-PAINT: Live cell Imaging using reVersible intEractions (LIVE) point accumulation for imaging in nanoscale topography (PAINT) is an *in vivo* super-resolution microscopy technique used to localize and track proteins over time with a resolution of around 20 nm. The protein of interest is fused to a short peptide sequence that is reversibly bound by a peptide-binding protein fused to a fluorescent protein, causing a transient immobilization that is visible as a transient fluorescent spike.

LOV: Protein tags with a light-oxygen-voltage (LOV) sensing domain bind flavin mononucleotide (FMN) or other blue-light-absorbing flavin chromophores that emit green fluorescence upon photoexcitation with blue or ultraviolet light without the requirement for oxygen.

MaP: Max-Planck-Probes. Modification of rhodamine-based dyes by replacing the carboxyl group with an electron-deficient amide resulted in fluorogenic dyes that show a fluorescence increase up to 1,000-fold upon binding to SLE tags. These cell-permeable dyes, which span the entire spectrum of the visible light were developed by the researchers in the Department of Chemical Biology at the Max Planck Institute for Medical Research and hence named Max-Planck-Probes (MaP).

MINFLUX: Minimal photon flux (MINFLUX) nanoscopy is a microscopy technique that uses photoswitching and an excitation beam featuring an intensity minimum that serves as a reference coordinate to achieve localization precision of individual fluorophores of less than 5 nm.

NanoBRET: The NanoBRET assay is based on the proximity-dependent bioluminescence resonance energy transfer (BRET) from NanoLuc, which acts as an energy donor and is fused to the protein of interest, to a fluorescently labelled HaloTag, which is the energy acceptor and is fused to another protein, to identify protein-protein interactions in living cells.

Nanobody (Nb): Nanobodies, also known as variable heavy chain of heavy-chain only antibodies (VHH antibodies) or single-domain antibodies, are fragments derived from heavy-chain only antibodies found in camelids and sharks. Unlike conventional antibodies, they are around 10-times smaller (~15 kDa), more robust, and can fold in reducing environments, while maintaining a high antigen-binding specificity and affinity.

NanoLuc: The 19.1 kDa NanoLuc luciferase (NLuc) is derived from *Oplophorus gracilirostris* and uses the coelenterazine analog furimazine to produce a bioluminescence signal that is approximately 100-fold brighter than that of firefly luciferase.

PALM: Photoactivated localization microscopy (PALM) is a super-resolution microscopy technique that uses photoactivatable fluorescent proteins or fluorophores to achieve resolutions of 20 nm by stochastic activation and localization until bleaching.

Pep-Tag: The peptide-tag (Pep-Tag) consists of 15 amino acids forming an alpha-helix derived from the heptad repeat of the glycoprotein 41 of HIV and is recognized by specific nano- and chromobodies.

photo-ANA: The unnatural bifunctional amino acid 2-amino-5-diazirinylnonynoic acid (photo-ANA), which can be used for BONCAT, contains an alkyne group for visualization and enrichment by click chemistry, and a photoreactive warhead in the form of a diazirine group for photo-crosslinking with proximal proteins upon exposure to ultraviolet light.

PipB2: PipB2 is a SPI2-T3SS effector of *Salmonella* that contributes to the maturation of the *Salmonella*-containing vacuole (SCV) and the biogenesis of tubular extensions from the SCV, the *Salmonella*-induced filaments (Sifs), by interacting with the kinesin light chain, a subunit of the kinesin-1 motor complex.

PopD: The hydrophobic translocator subunit PopD of the T3SS of *Pseudomonas aeruginosa* assembles with PopB to form heterodimers in host cell membranes.

PYP-tag: The 14 kDa self-labeling protein tag PYP is derived from the photoactive yellow protein and binds by transthioesterification of Cys69 to coumarin derivatives containing a quencher that is removed upon enzyme binding or having fluorogenic properties. Alternatively, ketone-based ligands react with Cys69 to form a stable thioether bond.

RAW264.7: The murine macrophage-like cell line RAW 264.7 is derived from a tumor in a male mouse induced with the Abelson leukemia virus and represents currently one of the most commonly used myeloid cell lines.

ReAsH: The red fluorescent biarsenic dye ReAsH derived from resorufin binds covalently to tetracysteine-tagged proteins.

SCV: *Salmonella*-containing vacuole. After invasion or phagocytic uptake, *Salmonella* Typhimurium resides in a membrane-bound compartment called the *Salmonella*-containing vacuole (SCV). SCV biogenesis, which is dependent on a specific subset of SPI2-T3SS effector proteins, can be divided into three stages, the third of which is characterized by bacterial replication as the SCV acquires late endosomal markers but does not mature into a bactericidal compartment.

Secretion: The process of exporting intracellular, cytoplasmic molecules across phospholipid membranes into the environment, facilitated by specialized secretion systems.

SIF: *Salmonella-*induced filaments. S*almonella*-induced fusions of host endomembranes of different organellar origin with the SCV leads to the formation of tubular structures, termed *Salmonella-*induced filaments (SIF), which extend throughout the cell and support intracellular proliferation and survival by recruiting nutrients. While SIF are highly dynamic in the initial phase, SIF biogenesis, which is dependent on a subset of SPI2-T3SS effectors, is characterized by a conversion from single-membrane to double-membrane tubular structures.

SifA: Following translocation, the SPI2-T3SS effector protein SifA, which is required for the formation of *Salmonella*-induced filaments and the maintenance of the *Salmonella*-containing vacuole, is isoprenylated and S-acylated, allowing membrane anchoring of the effector.

SipA: The *Salmonella* invasion protein A (SipA) is an SPI1-T3SS effector protein of *Salmonella* that acts as an F-actin binding protein, enhancing bacterial invasion efficiency by promoting actin polymerization. Additionally, during later stages of infection, SipA, together with SPI2-T3SS effectors, modulates SCV morphology and perinuclear positioning.

SiiC: The outer membrane secretin SiiC is a TolC homologue that forms the outer membrane pore of the SPI4-T1SS in *Salmonella*.

SiiF: The inner membrane ATPase SiiF belongs to the family of ATP-binding cassette transporters and is an important component of the SPI4-T1SS in *Salmonella.*

SIM: Structured Illumination Microscopy (SIM) is based on the excitation of the sample with a non-uniform structured pattern of light and relies on the generation of interference patterns to produce a super-resolution image.

SseF: The SPI2-T3SS effector SseF is an integral membrane protein localized to the *Salmonella* containing vacuole (SCV) and *Salmonella*-induced filaments (SIF). SseF supports the formation of a replication permissive niche by inhibiting Rab1A-mediated autophagy, contributes to the positioning of the SCV to a juxtanuclear, Golgi-associated localization, and mediates the maturation of the SIF network.

SMLM: Single-molecule localization microscopy is an umbrella term for imaging techniques that generate a time-resolved super-resolution image by localizing only a sparse subset of individual fluorophores at a given time to define molecular trajectories.

SMT: Single-molecule tracking (SMT) is used to investigate the movement of individual fluorescently labeled molecules in living cells as a function of time, the so-called trajectory, providing dynamic and structural parameters such as diffusion coefficients or spatial distributions.

SNAP-tag: The self-labeling enzyme tag SNAP-tag was derived in 2003 from the DNA repair protein O^6^-alkylguanine-DNA alkyltransferase, which reacts specifically and covalently with benzylguanine derivatives.

SopB: The SPI1-T3SS effector protein SopB, which has phosphoinositide phosphatase activity and a GTPase binding domain, was shown to promote bacterial cell entry by modulating actin cytoskeleton reorganization, and to contribute to the subcellular positioning and biogenesis of the SCV.

SopE/SopE2: The SPI1-T3SS effectors SopE and SopE2 share 69% amino sequence identity. As guanine nucleotide exchange factors for the Rho-family GTPases RAC1 and CDC42, they mediate actin cytoskeleton rearrangements essential for the invasion process of *Salmonella.*

SptP: The 60 kDa protein SptP is a SPI1-T3SS effector of *Salmonella* that represses the Rho GTPases Rac1 and Csc42 to reverse cytoskeletal changes induced by other SPI1-T3SS effectors, SopE/SopE2. Additionally, SptP was shown to downregulate the pro-inflammatory response by inhibiting the mitogen-activated protein kinase pathway and to be involved in SCV biogenesis.

SPIEDAC: Stained alkynes or alkenes undergo a Strain-promoted inverse-electron–demand Diels–Alder cycloaddition (SPEDAC) with tetrazines under physiological conditions, which is used for biorthogonal site-specific labeling in living cells with unnatural amino acids.

SRM: Super-resolution microscopy (SRM) is an umbrella term for a class of optical microscopy techniques that operate with a spatial resolution that is below the diffraction limit.

SsaP: The low abundant SPI2-encoded protein SsaP modulates the secretion specificity of the T3SS in *Salmonella* by mediating the transition from the secretion of substrates for injectisome (T3SS) assembly to the secretion of SPI2 effectors.

STED: In stimulated emission depletion (STED) microscopy, a first pulse is used to excite a fluorescent probe, followed by a second pulse to reversibly de-excite any fluorophore surrounding the excitation center, allowing imaging beyond the diffraction limit.

SunTag: The SunTag antibody-peptide labeling strategy is based on the recruitment of up to 24 copies of GFP-tagged antibodies, expressed in the host and directed against the short SunTag epitope fused to the protein of interest, for signal amplification.

SLE tags: Self-labeling enzymes (SLE) are proteins that catalyze high-affinity reactions with specific substrates that can be coupled to fluorochromes for specific fluorescent labeling of fusion proteins in living cells. The availability of different ligands for common SLE tags, such as HaloTag, SNAP-Tag, and CLIP-Tag, provides high labeling flexibility in terms of spectral properties, fluorogenicity, cell permeability, etc.

SPI1: The *Salmonella* pathogenicity island 1, which is required for host cell invasion and regulation of the host immune response, is a 40 kb gene cluster encoding a T3SS, the associated effector proteins, and chaperones as well as some transcriptional regulators that control the expression of virulence factors.

SPI2: *Salmonella* pathogenicity island 2 (SPI2) genes, required for intracellular survival and replication of *Salmonella* inside the host, are induced by acidic pH and low inorganic phosphate concentrations as encountered in the phagosome. SPI2 encodes a T3SS, several associated effectors and chaperones, and the two-component system SsrAB, involved in the regulation of SPI2 gene expression.

T3SS: The bacterial T3SS injectisome is a multimeric protein complex, that is evolutionary related to the flagella systems and is used to translocate of virulence proteins directly into the host cell. The T3SS consists of more than 20 different proteins, that form a needle-like structure spanning three membranes, with an inner needle diameter of 20-35 Å (2-3 nm), requiring the translocation of unfolded effector proteins.

T4SS: The T4SS, which shows similarities to the conjugation pili, functions in the delivery of unfolded effector proteins and in some cases DNA directly into host cells. In Gram-negatives, T4SS are characterized by a conserved structure, consisting of at least 12 subunits that form the cytoplasmic ATPase, the outer-membrane core complex connected to the inner membrane complex by a stalk and, in some species, the extracellular pili.

T6SS: The T6SS is the most recently discovered translocation system, primarily involved in interbacterial competition, but also in bacterial invasion and intracellular survival. Upon contact with the target cells, a rapid contraction of the sheath leads to the transfer of the tube through the membrane complex, that spans the entire bacterial envelope, and perforation of the target membrane by the spike complex and simultaneous effector delivery.

TALM: The super-resolution fluorescence microscopy technique, tracking and localization microscopy (TALM), allows the analysis of the spatio-temporal molecule dynamics by localizing and recording single trajectories of molecules labeled with photostable rhodamine dyes covalently bound to SLE tags at high frame rates with a precision of less than 20 nm until they are bleached.

TASEC: Tag-assisted split enzyme complementation (TASEC) approach is based on the fusion of the effector of interest to two orthogonal, short peptide tags (GFP11 and SpyTag), recognized by their respective binders (GFP1-10 and SpyCatcher), to promote complementation of fused split enzyme fragments (split HaloTag) for subsequent labeling in living cells.

TEM: Transmission electron microscopy (TEM) is an imaging technique that achieves nanometer resolution of the inner structure of a specimen by transmitting an electron beam through an ultrathin section of the fixed sample, producing an electron shadow depending on the local electron density.

Translocation: Effector translocation (or injection) describes the process of bacterial protein delivery by dedicated secretion systems across bacterial and host membrane directly into the target cell.

TMBDMA: The dialkylaminocoumarin derivative TMBDMA is non-fluorescent in a polar environment, but upon binding to the low-polar cavity of the PYP-tag, fluorescence increases significantly.

TMR: Tetramethylrhodamine is a rhodamine dye characterized by the xanthene ring system, an emission wavelength of 550-600 nm, and a high molar extinction coefficient of about 1 × 10^5^ M^-1^ cm^-1^. This widely used fluorophore can be coupled to ligands specific for self-labeling enzyme tags, such as HTL, and is applied in fluorescence microscopy, super-resolution microscopy (SRM), or due to its ability to photooxidize diaminobenzidine to an osmiophilic polymer, in electron microscopy (EM).

VirB/VirD4 T4SS: The T4SS of *Agrobacterium tumefaciens,* which is involved in the interkingdom transfer of T-DNA and effector proteins, consists of eleven VirB proteins (VirB1-VirB11), which form the secretion machinery and pilus, and the ATPase VirD4, which is responsible for substrate recruitment.

VirE2: The effector VirE2 of *Agrobacterium tumefaciens* is required for efficient T4SS-dependent plant transformation. By binding to the transferred ssDNA, VirE2 is the major component of the T-complex and is involved in several stages of the transformation process, including T-DNA entry, protection from nucleolytic degradation, nuclear targeting, and genome integration.

YopD: In *Yersinia,* translocator-dependent pore formation is mediated by YopD, YopB and LcrV. The translocon component YopD is also involved in the regulation of effector synthesis and secretion.

YopP: As a homologue of YopJ, the protease YopP belongs to the YopJ effector family and is involved in the downregulation of adhesion molecules, pro-inflammatory cytokines, the inflammasome formation through inhibition of the nuclear factor-κB and mitogen-activated protein kinase pathways in host cells.

YopJ: The evolutionary conserved T3SS-dependent Yersinia outer protein J (YopJ) effector family is widely distributed in plant and animal pathogens and is characterized by a conserved catalytic triad. Many YopJ family effectors, when activated by the host ligand inositol hexakisphosphate, possess acetyltransferase activity as an important virulence strategy to suppress pro-inflammatory signaling pathways and innate immunity in host cells.

xHTL: Exchangeable HaloTag ligands, termed xHTL, are cell-permeable derivatives of covalent HTL in which the chloride leaving group has been substituted with sulfonamides to create ligands that bind reversibly to the HaloTag 7 protein with dissociation constants in the nanomolar range. Coupled to rhodamines of different colors, xHTL can be applied for super-resolution microscopy where probe exchange reduces the risk of photobleaching and allows extended multi-frame and dual color-imaging.
